# SBCS-Net: Sparse Bayesian and Deep Learning Framework for Compressed Sensing in Sensor Networks

**DOI:** 10.3390/s25154559

**Published:** 2025-07-23

**Authors:** Xianwei Gao, Xiang Yao, Bi Chen, Honghao Zhang

**Affiliations:** Beijing Electronic Science and Technology Institute, Beijing 100070, China; 20233910@mail.besti.edu.cn (X.Y.); 20232940@mail.besti.edu.cn (B.C.); 20222942@mail.besti.edu.cn (H.Z.)

**Keywords:** compressed sensing, sensor networks, sparse Bayesian learning, deep learning

## Abstract

Compressed sensing is widely used in modern resource-constrained sensor networks. However, achieving high-quality and robust signal reconstruction under low sampling rates and noise interference remains challenging. Traditional CS methods have limited performance, so many deep learning-based CS models have been proposed. Although these models show strong fitting capabilities, they often lack the ability to handle complex noise in sensor networks, which affects their performance stability. To address these challenges, this paper proposes SBCS-Net. This framework innovatively expands the iterative process of sparse Bayesian compressed sensing using convolutional neural networks and Transformer. The core of SBCS-Net is to optimize key SBL parameters through end-to-end learning. This can adaptively improve signal sparsity and probabilistically process measurement noise, while fully leveraging the powerful feature extraction and global context modeling capabilities of deep learning modules. To comprehensively evaluate its performance, we conduct systematic experiments on multiple public benchmark datasets. These studies include comparisons with various advanced and traditional compressed sensing methods, comprehensive noise robustness tests, ablation studies of key components, computational complexity analysis, and rigorous statistical significance tests. Extensive experimental results consistently show that SBCS-Net outperforms many mainstream methods in both reconstruction accuracy and visual quality. In particular, it exhibits excellent robustness under challenging conditions such as extremely low sampling rates and strong noise. Therefore, SBCS-Net provides an effective solution for high-fidelity, robust signal recovery in sensor networks and related fields.

## 1. Introduction

The rapid development of sensing technology has put forward higher requirements for data acquisition and processing systems. Modern sensor networks, especially those in environmental monitoring [1], remote sensing [2], and biomedical applications [3], generate massive amounts of data, posing a major challenge to traditional sampling and transmission capabilities. These networks are often deployed in resource-constrained environments and face many limitations, including low bandwidth, limited transmission capacity, real-time processing requirements, and low energy consumption for long-term operation. These factors have created huge bottlenecks in data processing and operational efficiency.

**Figure 1:**
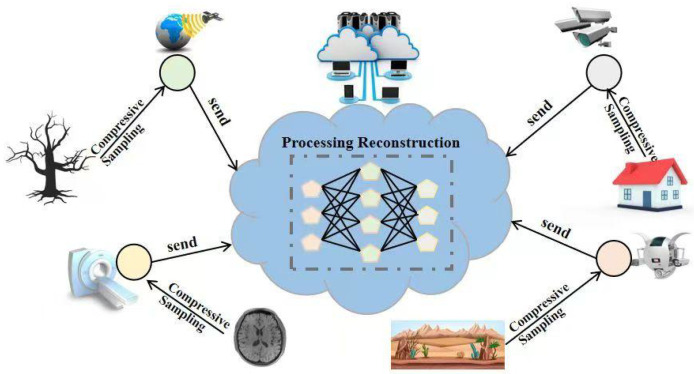
Sensor network.

Compressed sensing (CS) [4] is a breakthrough signal processing paradigm. It breaks away from the limitations of the Nyquist–Shannon sampling theorem [5] and provides a new approach to address the above challenges. By exploiting the sparse nature of signals in a specific domain, compressed sensing can achieve accurate signal reconstruction with far fewer measurements than traditional methods, making it an effective method for data acquisition and processing in many scientific and engineering fields [6,7]. In sensor networks, CS usually adopts an architecture of lightweight front-end sampling and complex back-end reconstruction. Its typical workflow is shown in Figure 1. Resource-constrained sensor nodes perform low-complexity compressed sampling through random projection or structured measurement to compress high-dimensional signals into low-dimensional space. This significantly reduces the amount of data transmission, bandwidth usage, and energy consumption. The computationally intensive reconstruction task is transferred to the cloud or edge computing nodes, using complex nonlinear algorithms to restore high-fidelity signals while meeting real-time requirements and avoiding sensor node overload. This division of labor effectively alleviates the bottlenecks in data transmission, energy consumption, and processing speed in resource-constrained sensor networks.

Although the theory of compressed sensing has made significant progress, its practical application still faces severe challenges, especially how to achieve sufficient reconstruction accuracy and robustness under conditions such as low sampling rate, complex signal structure, and noise interference. Traditional compressed sensing methods, whether based on convex optimization methods [8] or greedy algorithms, have obvious limitations. For example, the hyperparameter problem makes these traditional algorithms inefficient and low-precision, and they have strict requirements on signal sparsity and poor robustness. Although the early Bayesian compressed sensing (BCS) [9] method provides adaptive sparse learning and noise modeling from a probabilistic perspective to improve robustness, it has high computational complexity and is still sensitive to hyperparameters related to sparsity and noise precision. At the same time, deep learning-based methods solve the hyperparameter problem in traditional algorithms and achieve significant performance improvements through powerful nonlinear fitting, but their designs often focus on end-to-end mapping learning and network architecture design and may lack clear and adaptive probabilistic modeling mechanisms for signal sparsity and measurement noise. When encountering strong noise or extremely low sampling rates that are not fully reflected in the training data, it will lead to unstable performance or impaired generalization ability.

In order to make up for the lack of explicit probabilistic modeling in existing deep learning compressed sensing methods, make full use of the powerful feature extraction and efficient computing capabilities of deep neural networks, and give the compressed sensing reconstruction process stronger theoretical guidance, structured sparsity constraints and robust uncertainty handling capabilities, this paper proposes a deep Bayesian compressed sensing network (SBCS-Net). The core design concept of this network is that the SBL adaptive sparsity learning ability achieved by prior parameters and the probability-based noise modeling ability achieved by noise precision parameters can provide key regularization and effective guidance for deep learning models in sensor network environments with extremely sparse or severely polluted information. Specifically, this study aims to explore and clarify several key aspects of this fusion method. First, this study explores how to synergistically combine the adaptive sparsity enhancement and probabilistic noise modeling capabilities of sparse Bayesian learning (SBL) [10] with deep neural network architectures to overcome their inherent computing capabilities. Traditional SBL suffers from hyperparameter sensitivity, especially in resource-constrained sensor networks that typically operate at low sampling rates. Second, this study explores the potential of fusing the strengths of convolutional neural networks (CNNs) [11] in fine-grained local feature extraction with the complementary strengths of Transformer [12] in capturing global contextual information within an iterative SBL framework, aiming to significantly improve the reconstruction accuracy and robustness of complex signal structures. Finally, the core goal of this study is to determine whether such hybrid models can achieve a more ideal balance between reconstruction fidelity, computational efficiency, and adaptability to different noise conditions and signal types by learning key SBL parameters end-to-end, thereby providing a more robust and general solution for practical CS applications in challenging sensor network environments.

Instead of simply combining SBL with deep learning, SBCS-Net deeply integrates the core reasoning mechanism of SBL into an iterative deep architecture composed of CNN and Transformer, and designs key SBL parameters (such as α and β) as components that can be internally optimized through end-to-end learning. This design enables SBCS-Net to fully exploit the inherent advantages of SBL by learning its control parameters to adaptively improve signal sparsity and quantize/suppress noise—which is difficult to explicitly achieve in many purely data-driven deep learning models. At the same time, it overcomes the inherent limitations of SBL by reducing significantly its computational complexity by leveraging neural networks (especially image patch-based processing strategies and GPU parallel computing capabilities) and simplifying the parameter optimization process through end-to-end learning. In addition, the comprehensive performance of the deep learning module is also improved, because the fine-grained local detail capture of CNN and the excellent global dependency modeling of Transformer provide the SBL module with higher-quality signal representation, thereby achieving more accurate signal reconstruction under the guidance of the SBL probabilistic framework. The organic fusion of model-driven Bayesian principles and data-driven deep learning capabilities enables SBCS-Net to pursue computational efficiency and reconstruction accuracy while maintaining robustness and generalization potential that is superior to many purely data-driven deep learning models under challenging conditions.

Therefore, through this effective fusion of model-driven and data-driven approaches, SBCS-Net is dedicated to achieving superior compressed sensing performance under challenging conditions. The main contributions of this paper include the following:

- A novel compressed sensing framework, SBCS-Net, is proposed, which achieves a deep iterative integration of the core principles of SBL with deep neural networks (CNN and Transformer). Through a unique structural design, SBCS-Net effectively combines SBL’s adaptive sparse modeling, CNN’s local feature extraction, and Transformer’s global contextual awareness capabilities. It aims to achieve higher reconstruction accuracy and better noise robustness, particularly under challenging conditions such as low sampling rates and strong noise.
- Leveraging the end-to-end learning capability of neural networks, key hyperparameters from traditional BCS—such as those controlling sparsity and noise precision—are transformed into learnable parameters within the network. This design not only significantly alleviates the difficulty of hyperparameter optimization inherent in BCS but also effectively reduces the overhead of large-scale matrix computations, while retaining the theoretical advantages of Bayesian inference in uncertainty modeling and adaptive sparsity.
- The proposed model has undergone extensive experimental validation on multiple public datasets covering diverse scenarios and has been comprehensively compared with current mainstream deep learning CS methods. Preliminary experimental results indicate that the proposed method can achieve superior reconstruction quality and visual effects under various conditions, especially in cases of low sampling rates and noisy measurements.

The remainder of this paper is organized as follows: Section 2 reviews related work in CS reconstruction methods and deep learning approaches. Section 3 presents the detailed architecture and methodology of SBCS-Net. Section 4 provides experimental results and comparative analysis. Finally, Section 5 concludes with discussions on the implications of this work and future directions.

## 2. Related Work

This section gives a brief presentation of related works, i.e., conventional CS theory and its Bayesian extension, DL-based models for image CS reconstruction, and Transformer architectures for modeling global dependencies. These works form the foundation of our proposed SBCS-Net framework.

### 2.1. Traditional Compressed Sensing

Traditional CS methods can be primarily categorized into two main schools: convex optimization algorithms and greedy algorithms.

Convex optimization methods, such as Basis Pursuit (BP) [13], Iterative Soft-Thresholding Algorithm (ISTA) [14], and its accelerated versions (e.g., FISTA [15]), provide a solid mathematical foundation for CS problems by relaxing the NP-hard $l_{0}$-norm minimization problem into a more tractable convex $l_{1}$-norm minimization problem. For instance, under the condition that the sensing matrix satisfies the well-known Restricted Isometry Property (RIP) and when the signal is sufficiently sparse, BP can theoretically guarantee the exact recovery of sparse signals or achieve stable recovery in the presence of noise. The mathematical completeness of these methods established them as central to early CS theory. However, these approaches typically entail high computational complexity. BP, for example, may involve linear programming, with its complexity potentially reaching the order of $O(M^{2}N^{2})$ in some scenarios, or it may depend on a large number of iterations (as in ISTA/FISTA, where the cost per iteration is low, but the total number of iterations can be substantial), leading to high overall computational costs. Furthermore, their performance can degrade significantly when dealing with very low sampling rates or complex noise.

Greedy algorithms, such as Matching Pursuit (MP), Orthogonal Matching Pursuit (OMP) [16], and Subspace Pursuit (SP) [17], adopt an iterative strategy, selecting the atom (or set of atoms) most correlated with the current measurement residual at each step to progressively construct the sparse solution. This intuitive and efficient approach to signal approximation is their primary characteristic. The main operations in a single iteration of OMP include calculating correlations (approximately O(MN)) and solving a small-scale least-squares problem. If the signal sparsity is K, the total computational complexity is roughly on the order of O(KMN), thus generally being faster than convex optimization methods. However, their performance is highly dependent on the signal’s sparse structure and mathematical properties of the measurement matrix, such as the RIP or mutual coherence. When these conditions are not ideally met, greedy algorithms are more prone to getting trapped in local optima and may lack universality for complex signals.

Overall, while these early methods that laid the groundwork for CS theory pioneered signal recovery from undersampled data and provided mathematical guarantees for recovery and complexity bounds under specific conditions, their initial framework has gradually revealed inherent limitations in terms of methodological innovation and performance improvement when faced with the increasing demands for reconstruction quality (especially under non-ideal conditions), computational efficiency, and robustness.

### 2.2. Bayesian Compressed Sensing

To address certain limitations of traditional methods, BCS introduced a principled probabilistic modeling framework for the CS reconstruction problem. BCS models the reconstruction process using hierarchical Bayesian inference, providing a more comprehensive theoretical foundation for sparse signal recovery.

Its core lies in the probabilistic representation of the measurement process:

(1) y=Φx+ϵ

where the observation vector y is formed by the true signal x undergoing a linear transformation via the sensing matrix Φ and being superimposed with zero-mean Gaussian noise $\epsilon ∼N(0,\beta^{-1}I)$, where β is the noise precision. This explicit noise modeling is key for BCS to handle uncertainty and enhance noise robustness.

The key innovation of BCS lies in its hierarchical sparse prior structure. First, a zero-mean Gaussian prior is set for each component $x_{i}$ of the signal x (note: $x_{i}$ itself is a scalar component and thus not bolded):

(2) $$p\left(x_{i}|\alpha_{i}\right)=N\left(x_{i}|0,\alpha_{i}^{-1}\right)$$

where the precision $\alpha_{i}$ controls sparsity. Subsequently, a conjugate Gamma hyperprior is set for each $\alpha_{i}$:

(3) $$p\left(\alpha_{i}|a,b\right)=\mathrm{Gamma}\left(\alpha_{i}|a,b\right)$$

to achieve adaptive learning of sparsity. This hierarchical structure is central to the adaptivity of Bayesian models.

Based on this, the Gaussian posterior distribution of the signal x:

(4) p(x|y,α,β)=N(μ,Σ)

can be inferred via Bayes’ theorem, with its mean μ and covariance Σ given by:

(5) $$\begin{array}{cc} \mu & =\beta \Sigma \Phi^{T}y \end{array}$$

(6) $$\begin{array}{cc} \Sigma & ={\left(\beta \Phi^{T}\Phi +\mathrm{diag}\left(\alpha \right)\right)}^{-1} \end{array}$$

where α in diag(α) typically denotes the vector of individual precision parameters ${\left[\alpha_{1},\ldots ,\alpha_{N}\right]}^{T}$.

This probabilistic treatment endows BCS with several theoretical advantages: Automatic Relevance Determination (ARD) is achieved by inferring hyperparameters $\alpha_{i}$ to prune irrelevant components; the posterior covariance Σ provides principled uncertainty quantification; and the integration of the noise parameter β allows for more flexible handling of observation noise, thereby theoretically equipping BCS with good noise robustness. The emergence of BCS undoubtedly provided the CS field with a more intelligent solution paradigm, distinctly different from traditional optimization.

However, BCS also faces challenges in practical applications: the $O(N^{3})$ matrix inversion involved in solving for the posterior covariance Σ makes it difficult to scale to large-scale signals; its performance is highly dependent on the precise estimation of model hyperparameters α (referring to the vector of all $\alpha_{i}$’s or their common governing parameters) and β, which often requires complex optimization or manual tuning in traditional BCS; and commonly used Gaussian priors may not adequately capture the complex sparse characteristics of real signals. These factors indicate that although the probabilistic framework of BCS offers profound insights for CS research, critical breakthroughs are still needed in how to apply this framework efficiently, robustly, and conveniently to diverse practical problems, especially regarding computational feasibility, automatic hyperparameter optimization, and complex prior representation. This also clarifies the direction for subsequent research, including the SBCS-Net proposed in this paper.

### 2.3. Deep Compressed Sensing

In recent years, deep learning has brought great progress to the field of CS reconstruction, and its powerful nonlinear mapping ability has significantly improved the quality and application potential of reconstructed images. At present, DL-based compressed sensing methods can be roughly divided into two major directions: deep unfolding networks and end-to-end learning networks. Although these methods have made great progress, they still face continuous challenges and optimization needs in key aspects such as feature extraction efficiency, effective retention and transmission of information during iteration, performance stability under low sampling rates, and model generalization ability and noise robustness.

The core concept of deep unfolding networks is to cleverly transform the iterative solution steps of traditional optimization algorithms into a specific hierarchical structure in neural networks. This strategy effectively combines model-driven theoretical guidance with data-driven parameter learning. By optimizing key internal network parameters (such as iteration step size, sparsity threshold, and various learnable transformation modules) through end-to-end training, these networks can usually achieve better reconstruction performance and faster convergence speed than the original traditional algorithms, while retaining a certain degree of interpretability based on optimization theory. Many representative works have emerged in this direction. For example, ISTA-Net [18] and its subsequent versions are based on the concept of proximal gradient descent, expanding the iterative process of the ISTA algorithm and replacing the manually set sparsity constraints with learned nonlinear transformations; FSOINet [19] optimizes in the feature space. Unfolding networks based on the ADMM algorithm, such as ADMM-CSNet [20] and ADMM-Net, improve the performance of the original algorithm by introducing learnable parameters. AMP-Net [21], based on the AMP algorithm, focuses on solving noise and reducing block effects, while DRCAMP-Net [22] expands the receptive field by merging residual convolutions. In addition, other unfolding methods include NeumNet [23], which uses the Neumann series to solve the inverse problem; TransCS, which combines the ISTA-based Transformer backbone network with a CNN module for ICS reconstruction; and OCTUF [24], which draws on the classic PGD method and iterates the Transformer with a cross-attention mechanism. In order to further improve the convergence speed and modeling capabilities, some studies have begun to explore the combination of more advanced optimization strategies with novel sequence modeling ideas. SSM-Net [25] deeply integrates and unfolds the FISTA algorithm with the dependency modeling function of the Mamba-based state-space model (SSM), aiming to efficiently capture short-range and long-range signal dependencies with linear complexity through SSM, while accelerating convergence using the momentum mechanism of FISTA. Despite the significant progress made in deep unfolding networks, as USB-Net [26] pointed out in their research, many existing unfolding methods still face some common problems, such as the lack of information exchange in the iterative reconstruction stage, which leads to inefficient feature extraction and information loss, which is particularly evident at low sampling rates, i.e., the quality of details in the reconstructed image is reduced. This is also one of the core challenges that our current research, SBCS-Net, attempts to solve by deeply integrating the Bayesian mechanism with advanced feature extraction and fusion modules.

Unlike deep unfolding networks, end-to-end learning networks directly construct complex nonlinear mappings from compressed measurements to original signals without explicitly relying on the framework of traditional optimization algorithms. These methods take full advantage of powerful feature learning and end-to-end optimization capabilities of deep neural networks. Among them, methods based on CNN are a primary focus of research. The early ReconNet [27] pioneered the application of CNN in CS recovery. Subsequent models such as CSNet+ (which learns both the sampling pattern and reconstruction), DeepCodec [28], DR2-Net [29] (which employs residual learning for improved reconstruction), and DPA-Net [30] (which uses dual-path attention to separately capture structural and texture information) have further enhanced reconstruction performance. However, the inherent local receptive field of standard CNN limits their ability to effectively capture global image dependencies. To address this, methods like MSCRLNet [31] employ multi-scale design and residual learning strategies to expand the effective receptive field. To more directly model long-range dependencies, researchers have introduced the Transformer architecture, initially applied in natural language processing, into the CS domain. TCS-Net, for instance, is a Transformer-based hierarchical framework that achieves reconstruction from patch-wise outlines to pixel-wise textures. Although Transformer excels at global feature modeling due to its self-attention mechanism, its core self-attention operation typically has a quadratic computational complexity ($O(n^{2})$) related to the input sequence length, which may limit its application in scenarios with high demands on computational efficiency. Furthermore, methods based on Generative Adversarial Networks (GANs), such as CSGAN, Task-aware GAN [32], and Sub-pixel GAN [33], have shown potential in enhancing the visual realism and detail richness of reconstructed images by introducing adversarial learning mechanisms, but their training stability and the fidelity of generated content areas remain of concern. Other representative model-driven approaches, such as AutoBCS [34] and MAC-Net [35], have also contributed to this field. In general, these end-to-end or purely model-driven deep learning methods often surpass traditional algorithms in reconstruction speed and peak metrics and can automatically learn parameters from data. However, their main limitations lie in their “black-box” nature, lacking clear interpretability rooted in CS theory, and their performance is highly dependent on the quantity and distribution of training data. When faced with out-of-distribution data or extremely adverse measurement conditions, their generalization ability and robustness can be severely challenged.

In summary, deep learning technology has undoubtedly injected strong momentum into the field of compressed sensing reconstruction and achieved significant performance improvements. However, many current leading methods innovate more at the level of network architecture engineering or training strategies, rather than achieving profound insights into the inherent theories of compressed sensing, such as signal sparsity and uncertainty modeling, and deeply integrating these with the powerful representation capabilities of deep learning. There remains vast research space and urgent challenges in how to more fundamentally incorporate domain-specific prior knowledge from CS into deep networks, substantially enhance model generalization ability and robustness in real-world complex application environments, and achieve an optimal balance between pursuing excellent performance and managing computational efficiency and resource consumption.

### 2.4. Transformer

The Transformer [36] architecture, originally introduced for natural language processing, has demonstrated strong capabilities in capturing long-range dependencies due to its self-attention mechanism. In computer vision, Transformer reformulates images as sequences of flattened patches and processes them using multi-head self-attention (MHSA) and positional encodings. Let z represent one such vectorized image patch from the input sequence; for each input patch *z*, the positional encoding (PE) is added to retain spatial information:

(7) $$\mathrm{PE}\left(z\right):=\left\{\begin{array}{cc} \sin\left(\frac{z_{\mathrm{pos}}}{10,000^{2i/\dim}}\right) & \mathrm{if}\;i\;\mathrm{is}\;\mathrm{even},\\ \cos\left(\frac{z_{\mathrm{pos}}}{10,000^{2i/\dim}}\right) & \mathrm{if}\;i\;\mathrm{is}\;\mathrm{odd}, \end{array}\right.$$

where $z_{\mathrm{pos}}$ and *i* are the positional index and dimension index, respectively. This encoding allows Transformer to capture both spatial and semantic relationships across image patches.

In Transformer, the MHSA mechanism plays a key role in capturing dependencies among different patches. For each attention head *h*, the queries *Q*, keys *K*, and values *V* are computed as:

(8) $$\mathrm{Attention}\left(Q,K,V\right)=\mathrm{softmax}\left(\frac{QK^{T}}{\sqrt{d_{k}}}\right)V,$$

where $d_{k}$ is the dimension of each head. By using multiple heads, the model can focus on various aspects of the input sequence, providing a richer representation of the image.

In the field of image CS, combining Transformer with CNN can help capture global and local dependencies. For example, TransCS [37] uses a two-stream structure where Transformer and CNN are fused together to improve performance by leveraging the strengths of both architectures.

### 2.5. Application Scenario: SBCS-Net in Sensor Networks

The practical application of a sophisticated algorithm like SBCS-Net within sensor networks is enabled by a specific architectural paradigm: lightweight front-end sampling and complex back-end reconstruction. In this model, the resource-constrained sensor node performs only a computationally simple linear projection to compress the acquired signal. This step’s primary purpose is to drastically reduce the amount of data for wireless transmission, thus saving critical energy and bandwidth.

The compressed, low-volume data is then sent to a powerful back-end server, which is where SBCS-Net is deployed. On this server, our algorithm executes its intensive reconstruction process to recover a high-fidelity signal. This division of labor makes it practical to leverage advanced reconstruction models, as their computational complexity does not burden the sensor nodes. Instead, the superior performance of SBCS-Net at the back-end enhances the overall efficiency and data quality of the entire sensor network system.

## 3. Proposed Method

**Figure 2:**
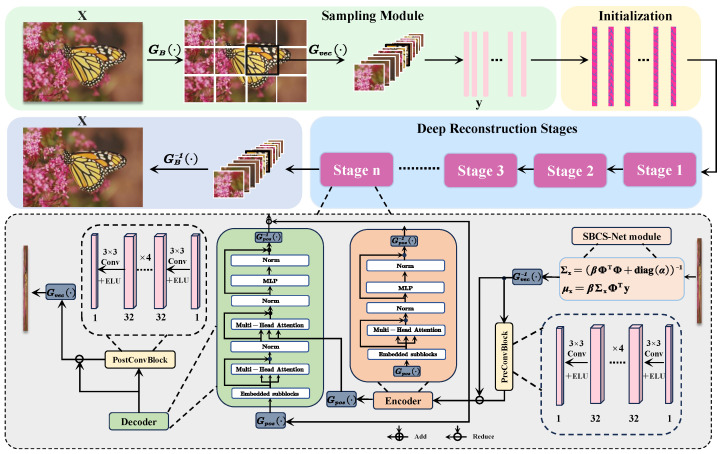
SBCS-Net framework.

This section will elaborate on the proposed SBCS-Net framework. SBCS-Net adopts a staged and modular design, aiming to decompose the complex compressed sensing reconstruction task into a series of ordered and interconnected processing steps, ultimately achieving efficient compressive sampling from the original signal and precise recovery of the final high-quality image. Figure 2 is the framework diagram of SBCS-Net, clearly illustrating its overall data flow.

The core workflow of this framework begins with the sampling module, which is responsible for acquiring low-dimensional compressed measurements from the original high-dimensional signal. These measurements are subsequently fed into an initial reconstruction stage, which utilizes a relatively simple mapping to quickly generate a preliminary estimate of the original signal. This preliminary estimate then serves as the input for the core deep reconstruction stage. The deep reconstruction stage employs an innovative iterative optimization mechanism, fusing Bayesian sparse estimation with deep learning techniques, to refine the signal through multi-level and systematic processing.

To ensure that all modules can be collaboratively optimized to achieve the best overall reconstruction performance, we have also designed a corresponding loss function for end-to-end network training. The following subsections will provide detailed explanations of these key components constituting SBCS-Net, along with their intrinsic working principles and optimization mechanisms.

### 3.1. Sampling Module

To achieve superior image reconstruction results, the sampling module of SBCS-Net utilizes a data-driven trainable sensing matrix. This module first divides the original image x into non-overlapping blocks of size B×B using a partitioning function $G_{B}\left(\cdot \right)$, and subsequently converts these image blocks into one-dimensional vectors using a flattening function $G_{vec}\left(\cdot \right)$. These vectorized image blocks are then linearly projected via a learnable sensing matrix $\Phi \in R^{M\times N}$. Here, $N=B^{2}$ denotes the original dimension of the vectorized image blocks, while $M=\lfloor \tau \times B^{2}\rfloor$ represents the dimension of the compressed measurements under a sampling rate τ=M/N (where 0≤τ≤1).

Unlike traditional methods that employ fixed random matrices, the sensing matrix Φ in this module is an integral part of the network, with its parameters optimized through an end-to-end learning process. Specifically, Φ is initialized according to a specific probability distribution before training commences and is iteratively updated throughout the training of the entire SBCS-Net network, along with all other learnable parameters, using the Adam optimizer with respect to the final reconstruction loss function. This data-driven optimization allows the sensing matrix Φ to automatically adapt to the intrinsic statistical properties of the training data, learning sampling patterns most conducive to information preservation and subsequent reconstruction; its final element distribution is the result of this optimization learning process. To more realistically simulate actual measurement scenarios, a Gaussian noise term ϵ is introduced during the sampling process to represent the inherent imperfections and uncertainties of the measurement system. Synthesizing these operations, the mathematical expression for the sampling module is as follows:

(9) $$y=S\left(x,\Phi \right)=\Phi \cdot G_{vec}\left(G_{B}\left(x\right)\right)+\epsilon$$

where S(·,Φ) represents the entire sampling process. Utilizing a learned sensing matrix, compared to random matrices, is generally more efficient for hardware implementation and requires fewer storage resources. It is noteworthy that SBCS-Net aims to obtain a universal sampling operator with good generalization capabilities across different signal types and common noise levels by learning a single sensing matrix Φ from diverse training data. Therefore, the parameters of Φ are fixed after training is complete. The model’s adaptation to the characteristics of specific input signals then primarily relies on the learnable noise precision parameter β within the Bayesian sparse estimation module in the subsequent deep reconstruction stage, as well as the powerful feature representation and generalization capabilities of the deep learning modules themselves. Furthermore, the explicit introduction of the noise term ϵ, controlled by the precision parameter β, not only enables the model to better handle noise present in actual measurements but also provides a basis for uncertainty quantification within the Bayesian framework. By actively considering the imperfections in real-world measurement processes, it effectively enhances the robustness of the subsequent reconstruction process.

According to Equation (9), this sampling process ultimately generates the compressed measurement vector y. This vector is not only a compact, low-dimensional representation of the original high-dimensional signal x but also constitutes the core observational data upon which all subsequent signal reconstruction stages must rely.

### 3.2. Initial Reconstruction Stage

After obtaining the compressed measurement vector y from the sampling module, the recovery process begins with the initial reconstruction stage. The objective of this stage is not to achieve a perfect reconstruction, but rather to provide an effective starting point for the subsequent deep iterative refinement. This is accomplished by applying a simple linear transformation that acts as an approximate inverse projection, mapping the low-dimensional measurements back to the high-dimensional signal space.

To perform this operation, we introduce an initial reconstruction matrix ${\tilde{\Phi }}^{T}\in R^{N\times M}$. Crucially, this matrix is not a fixed pseudo-inverse. Instead, its parameters are learned end-to-end along with the rest of the network. The sensing matrix Φ used in the sampling module is also optimized during training and then fixed during inference. The reconstruction matrix ${\tilde{\Phi }}^{T}$, however, is specifically trained to produce the most beneficial initial estimate for the subsequent stages. This data-driven approach allows the network to find an initial mapping that is superior to a simple, fixed transpose operation. The resulting preliminary estimate, $\mu_{x}^{0}$, is calculated as:

(10) $$\mu_{x}^{0}={\tilde{\Phi }}^{T}y$$

Due to the inherent information loss during the compressed sensing process, the preliminary estimate $\mu_{x}^{0}$ obtained from this approximate inverse is naturally coarse and typically exhibits reconstruction artifacts, such as noise amplification and loss of structural details.

Despite these limitations, $\mu_{x}^{0}$ serves a critical purpose. Rather than being a final output, it is merely an iterative starting point for the deep reconstruction stages. A well-designed initial estimate provides preliminary structural information—superior to a random or zero initialization—that helps guide the convergence of the subsequent iterative optimization process and improves its efficiency. Therefore, this initial reconstruction is a necessary foundation for the complex refinement process that follows.

### 3.3. Deep Reconstruction Stage

The deep reconstruction stage constitutes the core component of the SBCS-Net model. The design objective of this stage is to achieve high-quality image reconstruction through a hybrid multi-stage iterative optimization process, progressively refining the signal. The overall workflow of this iterative framework follows the description in Algorithm 1, encompassing *n* optimization stages, with the iteration variable *k* ranging from 1 to *n*. For the first optimization stage (i.e., k=1), the input is the preliminary reconstructed signal $\mu_{x}^{0}$ as described in Section 3.2; for any subsequent stage *k* (where k>1), further refinement is performed based on the optimized signal output from the preceding stage k−1.
**Algorithm 1** Forward propagation for SBCS-Net.**Require:** Number of iterations *n*, measurements y, sensing matrix Φ, initial reconstruction matrix $\tilde{\Phi }$, trainable parameters {α,β}
**Ensure:** Reconstructed image $\hat{x}$
  1:$\mu_{x}^{0}={\tilde{\Phi }}^{⊤}y$, k=1  2:**while** k≤n **do**  3:   $\Sigma_{x}^{k}={\left(\beta^{k}\Phi^{⊤}\Phi +\mathrm{diag}\left(\alpha^{k}\right)\right)}^{-1}$, $\mu_{x}^{k}=\beta^{k}\Sigma_{x}^{k}\mu_{x}^{k-1}$  4:   $x_{\mathrm{sbl}}^{k}=\mu_{x}^{k}$  5:   $x_{\mathrm{pre}}^{k}=G_{\mathrm{vec}}^{-1}\left(x_{\mathrm{sbl}}^{k}\right)-N_{\mathrm{pre}}^{k}\left(G_{\mathrm{vec}}^{-1}\left(x_{\mathrm{sbl}}^{k}\right)\right)$  6:   $x_{\mathrm{en}}^{k}=T_{\mathrm{en}}^{k}\left(G_{p}\left(x_{\mathrm{pre}}^{k}\right)+\mathrm{PE}\left(G_{p}\left(x_{\mathrm{pre}}^{k}\right)\right)\right)$  7:   $x_{\mathrm{de}}^{k}=x_{\mathrm{pre}}^{k}-T_{\mathrm{de}}^{k}\left(x_{\mathrm{pre}}^{k},x_{\mathrm{en}}^{k}\right)$  8:   $x_{\mathrm{post}}^{k}=x_{\mathrm{de}}^{k}-N_{\mathrm{post}}^{k}\left(x_{\mathrm{de}}^{k}\right)$  9:   $x_{\mathrm{fin}}^{k+1}=G_{\mathrm{vec}}\left(x_{\mathrm{post}}^{k}\right)$10:   k=k+111:**end while**12:$\hat{x}=G_{B}^{-1}\left(G_{\mathrm{vec}}^{-1}\left(x^{n+1}\right)\right)$


Within each independent optimization stage *k*, a structured signal processing architecture is employed. This architecture organically integrates the theoretical guidance of Bayesian sparse estimation with the powerful representation capabilities of deep learning. Specifically, the input signal for the current stage is first analyzed and updated for sparsity by the Bayesian sparse estimation module based on a probabilistic model; a detailed explanation of this module is provided in Section 3.3.1. Subsequently, the output from the Bayesian sparse estimation module is passed to a series of cascaded deep learning modules. These modules are responsible for deep feature extraction, complex pattern recognition, and the effective fusion and refinement of global and local information; their specific compositions and working mechanisms will be elucidated section by section from Section 3.3.2, Section 3.3.3, Section 3.3.4 and Section 3.3.5. The signal output mechanism after processing by all modules in the current stage is described in Section 3.3.6.

#### 3.3.1. Bayesian Sparse Estimation

To enforce signal sparsity and handle measurement noise in a principled, model-driven way, each reconstruction stage begins with this module. It provides a probabilistically regularized signal for subsequent deep learning-based refinement. The core task of this module is to update the sparse representation of the signal in a probabilistic sense, by combining the reconstruction result $\mu_{x}^{k-1}$ from the previous stage (k−1) (or the initial reconstructed signal $\mu_{x}^{0}$ for k=1) and relying on the parameters $\alpha^{k}$ and $\beta^{k}$ learned at the current stage. This method is based on the probabilistic framework of BCS, which assumes that the measurement process is affected by Gaussian noise and endows the signal with a Gaussian sparsity prior to promote its sparse characteristics.

In SBCS-Net, the noise robustness and adaptive capability to noise of this Bayesian sparse estimation module are primarily achieved by designing the key hyperparameters from traditional BCS—namely, the precision vector α controlling sparsity and the noise precision β—as network-trainable parameters for the current stage *k* (denoted as $\alpha^{k}$ and $\beta^{k}$). These parameters are optimized through end-to-end training of the entire network, guided by the final reconstruction loss function. In particular, the learning mechanism for the noise precision parameter $\beta^{k}$ enables the model to automatically adjust its effective perception of and response strategy to the noise level in the observed data, based on the training data.

Under this probabilistic setting and parameter learning mechanism, the posterior distribution of the signal x at stage *k* can be approximated as a Gaussian distribution. Its posterior covariance matrix $\Sigma_{x}^{k}$ and posterior mean $\mu_{x}^{k}$ (referencing Algorithm 1) are calculated, respectively, as:

(11) $$\Sigma_{x}^{k}={\left(\beta^{k}\Phi^{⊤}\Phi +\mathrm{diag}\left(\alpha^{k}\right)\right)}^{-1}$$

(12) $$\mu_{x}^{k}=\beta^{k}\Sigma_{x}^{k}\mu_{x}^{k-1}$$

In these formulas, the learned noise precision $\beta^{k}$ directly modulates the influence weight of information from the previous stage’s estimate $\mu_{x}^{k-1}$ (acting indirectly through $\Sigma_{x}^{k}$) in the calculation of the current posterior mean $\mu_{x}^{k}$. This means that when faced with higher noise (or greater uncertainty in the previous stage’s result), if the network learns a smaller value for $\beta^{k}$, it will reduce its reliance on potentially noisy data (or uncertain input) and depend more on the sparse structural information implied by the Gaussian prior, thereby suppressing noise. Conversely, if $\beta^{k}$ is larger, it places more trust in the data from the previous stage. This adaptive adjustment is key to improving the quality of signal recovery. The sparse estimate $x_{\mathrm{sbl}}^{k}$ for the current stage is this posterior mean:

(13) $$x_{\mathrm{sbl}}^{k}=\mu_{x}^{k}$$

This $x_{\mathrm{sbl}}^{k}$, as the output of the Bayesian sparse estimation module for the current stage, integrates information from the preceding stage, the currently learned parameters $\alpha^{k}$ and $\beta^{k}$, and the regularization effect of the probabilistic model. It provides a signal estimate that has undergone initial sparsity enhancement and noise consideration for the subsequent deep learning modules. By integrating the necessary matrix operations (such as the matrix inversion in Equation (11) and matrix-vector multiplications in Equation (12)) into the neural network’s computation graph and leveraging the optimization capabilities of the deep learning framework, this module avoids the separate and potentially more time-consuming iterative parameter solving process found in traditional BCS, thereby effectively managing computational complexity.

#### 3.3.2. Pre-Block: Local Feature Optimization

While the Bayesian estimate provides a good sparse signal, it may lack fine-grained textures or contain minor artifacts. The Pre-block is therefore introduced to perform initial local feature enhancement and artifact suppression using CNNs. After the sparse estimation signal $x_{sbl}^{k}$ output by the Bayesian sparse estimation module is transformed into an image domain representation via $G_{vec}^{-1}$, although it possesses theoretical sparse advantages, it may still be deficient in the depiction of local details, such as potential smoothing effects or slight artifacts. To solve this problem and provide high-quality input for subsequent global feature modeling, we send $G_{vec}^{-1}\left(x_{sbl}^{k}\right)$ into the Pre-block module. The core function of the Pre-block is to utilize the powerful local feature extraction and non-linear mapping capabilities of CNN to perform initial local feature enhancement and artifact suppression on $G_{vec}^{-1}\left(x_{sbl}^{k}\right)$. This is an initial manifestation of the synergistic effect between Bayesian sparse estimation results and the local perception capabilities of CNN, aiming to improve the quality of the signal representation. After the sparse estimation signal $x_{sbl}^{k}$ output by the Bayesian sparse estimation module is transformed into an image domain representation via $G_{vec}^{-1}$, although it possesses theoretical sparse advantages, it may still be deficient in the depiction of local details, such as potential smoothing effects or slight artifacts. To solve this problem and provide high-quality input for subsequent global feature modeling, we send $G_{vec}^{-1}\left(x_{sbl}^{k}\right)$ into the Pre-block module. The core function of the Pre-block is to utilize the powerful local feature extraction and non-linear mapping capabilities of CNN to perform initial local feature enhancement and artifact suppression on $G_{vec}^{-1}\left(x_{sbl}^{k}\right)$. This is an initial manifestation of the synergistic effect between Bayesian sparse estimation results and the local perception capabilities of CNN, aiming to improve the quality of the signal representation.

This module usually consists of several convolutional layers, batch normalization layers, and non-linear activation functions. Its operation at stage k can be represented as a residual learning process, and the Pre-block operation is expressed as:

(14) $$x_{pre}^{k}=G_{vec}^{-1}\left(x_{sbl}^{k}\right)-N_{pre}^{k}\left(G_{vec}^{-1}\left(x_{sbl}^{k}\right)\right)$$

where $N_{pre}^{k}\left(\cdot \right)$ represents the CNN network of the Pre-block at stage k. By learning and subtracting the correction term $N_{pre}^{k}\left(G_{vec}^{-1}\left(x_{sbl}^{k}\right)\right)$, the network is guided to focus on identifying and eliminating local defects in the input signal, such as residual noise, blurred edges, or block effects, while simultaneously enhancing useful textures and structural details. This design enables the Pre-block to effectively purify the results of Bayesian sparse estimation. Its output, $x_{pre}^{k}$, is a clearer and more informative signal version at the local feature level, laying a good foundation for the global context analysis sent to the Transformer encoder.

#### 3.3.3. Encoder: Global Feature Modeling

A purely local approach struggles with capturing the long-range dependencies essential for overall structural integrity. Although the signal $x_{pre}^{k}$, whose local features have been optimized by the Pre-block, exhibits enhanced local details, its representation may still be primarily confined to neighborhood information. To capture long-range dependencies and the overall structure among various regions in the image, we input $x_{pre}^{k}$ into the Transformer encoder module $T_{en}^{k}$. While the Bayesian sparse estimation and Pre-block modules primarily focus on the signal’s sparsity and local characteristics, the encoder introduces a powerful global feature modeling mechanism to SBCS-Net. This is a key step in the deep fusion of Bayesian methods, CNN, and Transformer to capture a broader context. Although the signal $x_{pre}^{k}$, whose local features have been optimized by the Pre-block, exhibits enhanced local details, its representation may still be primarily confined to neighborhood information. To capture long-range dependencies and the overall structure among various regions in the image, we input $x_{pre}^{k}$ into the Transformer encoder module $T_{en}^{k}$. While the Bayesian sparse estimation and Pre-block modules primarily focus on the signal’s sparsity and local characteristics, the encoder introduces a powerful global feature modeling mechanism to SBCS-Net. This is a key step in the deep fusion of Bayesian methods, CNN, and Transformer to capture a broader context.

Before entering the encoder, the input signal $x_{pre}^{k}$ is typically first segmented into a sequence of image patches via a patch partitioning operation $G_{p}\left(\cdot \right)$. Since the Transformer’s self-attention mechanism itself does not directly perceive the order or spatial position of elements in a sequence, positional encoding (PE) needs to be added to each image patch to incorporate spatial information into the representation. Let $Z^{0}=G_{p}\left(x_{pre}^{k}\right)+\mathrm{PE}\left(G_{p}\left(x_{pre}^{k}\right)\right)$ denote this initial sequence of patch embeddings with positional information.

The sequence $Z^{0}$ is then fed into the Transformer encoder $T_{en}^{k}$, which is composed of *L* identical encoder layers stacked sequentially. Each encoder layer, say layer *l* (where *l* ranges from 1 to *L*), takes the output $Z^{l-1}$ from the previous layer (with $Z^{0}$ being the input to the first layer) and produces an output $Z^{l}$. The core components of each encoder layer are an MHSA module and a feed-forward network (FFN), with layer normalization (LN) and residual connections applied for stable training and effective representation. Schematically, the operations within the *l*-th encoder layer can be expressed as:

(15) $$\begin{array}{cc} Z_{l}^{\prime } & =\mathrm{LN}(Z^{l-1}+\mathrm{MHSA}\left(Z^{l-1}\right)) \end{array}$$

(16) $$\begin{array}{cc} Z^{l} & =\mathrm{LN}(Z_{l}^{\prime }+\mathrm{FFN}\left(Z_{l}^{\prime }\right)) \end{array}$$

The final output of the entire encoder stage, after *L* such layers, is $X_{en}^{k}=Z^{L}$. Thus, the overall encoder operation can be summarized as (reiterating your original Equation (17) for clarity in this new context):

(17) $$X_{en}^{k}=T_{en}^{k}\left(Z^{0}\right)=T_{en}^{k}\left(G_{p}\left(x_{pre}^{k}\right)+\mathrm{PE}\left(G_{p}\left(x_{pre}^{k}\right)\right)\right)$$

The MHSA module is crucial for modeling the relationships among all elements in the input sequence $Z^{l-1}$. It achieves this by parallelly computing multiple self-attention “heads”, allowing the model to jointly attend to information from different representation subspaces at different positions. Each attention head operates on query (*Q*), key (*K*), and value (*V*) matrices, which are typically derived from the input $Z^{l-1}$ through learnable linear projections: $Q=Z^{l-1}W_{Q}$, $K=Z^{l-1}W_{K}$, $V=Z^{l-1}W_{V}$, where $W_{Q},W_{K},W_{V}$ are parameter matrices. The core computation for a single attention head is the scaled dot-product attention, given by:

(18) $$\mathrm{Attention}\left(Q,K,V\right)=\mathrm{softmax}\left(\frac{QK^{T}}{\sqrt{d_{k}}}\right)V$$

where $d_{k}$ is the dimension of the key vectors. The outputs of multiple such heads are then concatenated and linearly projected to produce the final MHSA output.

The encoder, by stacking these layers, can thereby dynamically capture the interdependencies among all regions in the input image, unrestricted by spatial distance. Its output, $X_{en}^{k}$, is a feature representation that deeply encodes global contextual information, containing rich semantics of each image patch and their interrelations within the overall image structure. This provides crucial global perspective guidance for the subsequent decoder reconstruction.

#### 3.3.4. Decoder: Signal Reconstruction to Image Space

To synthesize a coherent image, the abstract global context must be effectively combined with concrete local details. The features $X_{en}^{k}$, rich in global context and generated by the Transformer encoder (as detailed in Section 3.3.3), along with the Pre-block output $x_{pre}^{k}$ (which serves as a reference for local details), are jointly processed by the Transformer decoder module $T_{de}^{k}$. The primary task of the decoder is to effectively fuse the abstract high-level features $X_{en}^{k}$ with the concrete low-level features present in $x_{pre}^{k}$, and to progressively map this combined information back to a structured image space. This aims to generate an optimized reconstruction $x_{de}^{k}$ that balances both global consistency and local detail. The features $X_{en}^{k}$, rich in global context and generated by the Transformer encoder (as detailed in Section 3.3.3), along with the Pre-block output $x_{pre}^{k}$ (which serves as a reference for local details), are jointly processed by the Transformer decoder module $T_{de}^{k}$. The primary task of the decoder is to effectively fuse the abstract high-level features $X_{en}^{k}$ with the concrete low-level features present in $x_{pre}^{k}$, and to progressively map this combined information back to a structured image space. This aims to generate an optimized reconstruction $x_{de}^{k}$ that balances both global consistency and local detail.

Similar to the encoder architecture, $T_{de}^{k}$ typically consists of multiple stacked decoder layers. Each decoder layer usually incorporates a masked self-attention module (operating on representations derived from $x_{pre}^{k}$ and its own intermediate states), a cross-attention module (which attends to the encoder’s output $X_{en}^{k}$), and an FFN. LN and residual connections are also employed within these layers.

The Transformer decoder network, denoted as $T_{de}^{k}\left(x_{pre}^{k},X_{en}^{k}\right)$, thus takes both the local feature map $x_{pre}^{k}$ and the global context $X_{en}^{k}$ as inputs. It produces an output signal which represents the learned enhancement or refinement. Consistent with the additive residual connection shown in Figure 2, the output of the decoder stage, $x_{de}^{k}$, is formulated by adding this learned refinement back to the original Pre-block output:

(19) $$x_{de}^{k}=x_{pre}^{k}+T_{de}^{k}\left(x_{pre}^{k},X_{en}^{k}\right)\;$$

In this manner, the decoder effectively combines the long-range dependencies and global structural information captured by the encoder (via $X_{en}^{k}$ and processed by $T_{de}^{k}$) with the local feature details preserved in and passed through $x_{pre}^{k}$ (via the skip connection). This step is crucial for ensuring that the reconstructed signal possesses an accurate global layout and clear local content. Its output, $x_{de}^{k}$, is thus a more refined signal version in the image space, modulated and enhanced by global information, and serves as the input to the subsequent Post-block.

#### 3.3.5. Post-Block: Final Refinement and Output Stage

Although the signal $x_{de}^{k}$, obtained after processing by the Transformer decoder, has already fused global and local information and is of relatively high quality, there is still room for improvement in details, such as eliminating minor artifacts potentially introduced by complex transformations or sharpening edges. For this purpose, we introduce the Post-block module to perform final local feature optimization and quality enhancement on $x_{de}^{k}$. This module is structurally similar to the Pre-block and is also a CNN-based component. The Post-block can be regarded as the final fine-polishing step in the entire signal processing pipeline—following the deep fusion of sparsity guided by Bayesian sparse estimation, local details extracted by CNN, and global context captured by Transformer—to ensure the visual quality of the final output. The corresponding Post-block operation is represented as:

(20) $$x_{post}^{k}=x_{de}^{k}-C_{post}^{k}\left(x_{de}^{k}\right)\;$$

Here, $C_{post}^{k}$ represents the Post-block CNN network at the current stage *k*. $C_{post}^{k}$ learns to identify and remove any residual noise, artifacts, or undesired smoothness from $x_{de}^{k}$, while further sharpening image details and enhancing local contrast. The resulting $x_{post}^{k}$ is a visually clearer reconstruction with richer details and fewer artifacts, representing the best achievable reconstruction quality at the current optimization stage k, striking a good balance between global structural integrity and local detail fidelity.

#### 3.3.6. Final Output Stage: Reconstructed Signal Generation

After completing the iterative processing and refinement through Bayesian sparse estimation and a series of deep learning modules such as the Pre-block, Transformer encoder–decoder, and Post-block, the signal $x_{post}^{k}$ generated at the current optimization stage k already incorporates all optimization results from this stage. To format it for final image stitching or, if k<n, as input for the next iteration stage, $x_{post}^{k}$ is first processed by a vectorization operation $G_{vec}\left(\cdot \right)$. The corresponding operational expression is:

(21) $$x_{fin}^{k+1}=G_{vec}\left(x_{post}^{k}\right)\;$$

Here, $x_{fin}^{k+1}$ represents the vectorized signal that has undergone complete processing in stage k and is ready to enter stage k+1 or serve as the final output.

When all n optimization stages are completed, i.e., when the iteration variable k reaches n, the final, multi-stage deep-optimized vectorized signal $x_{fin}^{n+1}$ is obtained. To obtain the final visualized reconstructed image $\hat{x}$, this vectorized representation needs to be transformed back to the original complete image space through a two-step inverse mapping process. The corresponding operational expression is:

(22) $$\hat{x}=G_{B}^{-1}\left(G_{vec}^{-1}\left(x_{fin}^{n+1}\right)\right)\;$$

In this process, $G_{vec}^{-1}$ first reorganizes and rearranges the one-dimensional long vector, restoring it to block-wise two-dimensional image data. Subsequently, the $G_{B}^{-1}$ operation reassembles these image blocks according to their correct spatial positions in the original image, thereby generating the final complete image $\hat{x}$. These two transformation steps are crucial for maintaining the structural integrity and visual continuity of the reconstructed image, ensuring excellent visual quality.

### 3.4. Loss Function

SBCS-Net is an end-to-end framework designed to reconstruct the original input image x from its compressed measurements *y*, incorporating both sampling and reconstruction optimization within a unified architecture. The sampling module S(x,Φ) generates compressed measurements that serve as input to the reconstruction pipeline; this pipeline includes an initial coarse recovery via $I(y,\tilde{\Phi })$ followed by complex iterative hybrid reconstruction stages H(·). These reconstruction stages integrate sparsity-inducing constraints derived from Bayesian principles, particularly manifested in the adaptive handling of measurement uncertainty through the precision parameter β.

This framework employs a joint optimization strategy during training, simultaneously optimizing the sampling and reconstruction modules to minimize the difference between the reconstructed image ${\hat{x}}_{i}$ and its corresponding ground truth image $x_{i}$. By introducing the precision parameter β in the sampling process, the framework can adaptively balance measurement fidelity against noise robustness, ensuring that the final output ${\hat{x}}_{i}$, after all reconstruction stages are completed, achieves optimal fidelity in terms of both local details and global structure.

To quantify reconstruction quality and guide the optimization process, we employ the Mean Squared Error (MSE) [38] loss function. This function provides a robust measure of the pixel-wise difference between the reconstructed and ground truth images. The loss function for SBCS-Net is specifically expressed as:

(23) $$L=\frac{1}{2N}\sum_{i=1}^{N}\left|\right|{\hat{x}}_{i}-x_{i}{\left|\right|}_{2}^{2}\;$$

where $x_{i}$ denotes the *i*-th training sample, ${\hat{x}}_{i}$ represents its corresponding reconstructed output after all reconstruction stages are completed, and *N* indicates the total number of training samples. This differentiable objective function enables effective gradient-based optimization, thereby facilitating the collaborative optimization of the sampling and reconstruction modules, and enhancing the robustness and accuracy of image reconstruction by minimizing pixel-wise reconstruction error. The inclusion of the precision parameter β in the sampling process also serves as an implicit regularization for the reconstruction process, strengthening the framework’s ability to handle measurement uncertainties while maintaining high reconstruction fidelity.

It is noteworthy that there is a key interaction mechanism between the MSE loss function adopted by SBCS-Net and its core Bayesian sparse estimation module in the deep reconstruction stage. Firstly, through end-to-end training, the MSE loss directly drives the learning and optimization of internal parameters within the Bayesian module, such as sparsity precision and noise precision. This implies that these parameters are not set independently of the reconstruction task but are adjusted so that the output of the Bayesian module, after being processed by subsequent deep learning modules, can maximally improve the quality of the final reconstructed image under the MSE criterion. The strategy of integrating theoretical model parameters into a discriminative learning framework and optimizing them in a data-driven manner has achieved success in various model-unfolding-based deep learning methods, as exemplified in works like ISTA-Net and ADMM-Net. Secondly, the choice of the MSE loss function is theoretically compatible with the posterior mean estimation in Bayesian estimation, which targets the Minimum Mean Squared Error (MMSE). SBCS-Net, by optimizing for MSE overall, can be seen as learning a complex mapping to approximate this MMSE optimal estimate. Lastly, the Bayesian framework itself introduces beneficial inductive biases to the MSE-driven learning process. Through its inherent sparse priors and noise modeling, it helps guide the network towards more robust and structurally sound solutions, especially when dealing with under-sampled and noisy data. Therefore, the MSE loss and the Bayesian framework act synergistically in SBCS-Net to jointly enhance reconstruction performance.

### 3.5. Computational Complexity Analysis

The computational complexity of SBCS-Net is analyzed based on its operation over $N_{p}$ image blocks, each of size $N_{block}$, for *n* iterative reconstruction stages. The initial sampling and reconstruction phases primarily involve matrix-vector operations, with a complexity of approximately $O(N_{p}MN_{block})$, where *M* is the measurement dimension per block.

Within each of the *n* deep reconstruction stages, the Bayesian sparse estimation module requires an $O(N_{block}^{3})$ matrix inversion per block, leading to a dominant term of $O(N_{p}N_{block}^{3})$ for this module per iteration. The subsequent CNN-based Pre-block and Post-block modules, characterized by their depth and convolutional filter configurations, contribute a complexity proportional to $O(N_{p}\cdot C_{CNN})$, where $C_{CNN}$ represents the typical computational load of these CNN per block. Similarly, the Transformer-based encoder and decoder modules, with $L_{T}$ layers and processing sequences of length $N_{seq}$ (derived from each block) with an embedding dimension $d_{m}$, exhibit a complexity of roughly $O(N_{p}L_{T}N_{seq}\left(N_{seq}d_{m}+d_{m}d_{ff}\right))$, where $d_{ff}$ is the feed-forward network’s hidden dimension.

SBCS-Net achieves significant computational advantages over traditional BCS methods, which often face an $O(N_{img}^{3})$ cost for full-image matrix inversion ($N_{img}$ being total image pixels) and separate, costly hyperparameter optimization. These advantages stem from: (1) Block-wise processing, reducing the cubic complexity’s base to $N_{block}\ll N_{img}$. (2) End-to-end learning of SBL parameters ($\alpha^{k},\beta^{k}$) within *n* fixed stages, which bypasses traditional iterative hyperparameter inference. While the integrated deep learning modules introduce their own computational costs, the architecture is optimized for a practical balance between reconstruction efficacy and computational load. This positions SBCS-Net as a more scalable solution for CS applications.

## 4. Experimental Results

This section presents comprehensive experimental evaluations of the proposed SBCS-Net framework. We first describe the experimental settings, including dataset preparation, training details, and implementation specifications. Then, through extensive comparisons with state-of-the-art methods on multiple benchmark datasets, we demonstrate the superior reconstruction results of SBCS-Net. Furthermore, we analyze the framework’s robustness under various noise conditions and evaluate its computational complexity. Finally, we perform detailed ablation studies to validate the effectiveness of each key component in our architecture.

### 4.1. Experimental Settings

The training data for SBCS-Net is primarily based on the BSD500 [39] dataset. Renowned for its extensive content and diverse features, this dataset provides a solid foundation for the model to address various sensor network applications. Its richness helps SBCS-Net learn visual features for specific scenarios (e.g., environmental monitoring) and general image priors, thereby enhancing its capability to process diverse sensor data, including remote sensing, urban surveillance, and even some biomedical imagery. Therefore, our strategy of integrating all subsets of BSD500 (training, validation, and testing sets) for training, combined with data augmentation, aims to enable SBCS-Net to learn highly generalizable feature representations and reconstruction methods, thereby enhancing its practicality and robustness in real-world complex visual signal environments. For each original image, we first normalize its pixel values to the [0, 1] range and then randomly crop 200 patches of 96×96 pixels. This process generates a total of 100,000 training sample patches. To further enhance data diversity and improve the model’s generalization ability, we apply data augmentation techniques to these image patches, primarily including random horizontal and vertical flips, small-angle rotations, and scale adjustments. All preprocessed image patches are finally converted into single-channel grayscale image tensors. Set11 [40] is used as an independent validation dataset during this process. Final performance evaluation is conducted on multiple public benchmark datasets, including BSD100 [41], Set5 [42], Urban100 [43], UCMerced [44], and BrainImages. These diverse datasets encompass various image types such as natural scenes, urban landscapes, remote sensing, and medical images, broadly representing the visual data characteristics that various sensors might acquire in practical applications, thus facilitating a comprehensive assessment of SBCS-Net’s performance and generalization capabilities.

The model’s implementation follows typical deep learning compressed sensing configurations comparable to methods like ISTA-Net. Key parameters are set as follows: patch dimension P = 8, initial step size is 1.0, and the number of iterations H = 8. During the training phase, two core Bayesian parameters are set as learnable: α=1.0 for regulating image sparsity, and β=1.0 for modeling measurement noise. These parameters, along with the weights of other network parts, are jointly optimized via backpropagation within the training framework. Training is conducted for a total of 200 epochs with a batch size of 64. The initial learning rate is set to 0.001, with periodic decay between epochs 101 and 150, followed by a fixed learning rate for the final 50 epochs. We employ the Adam optimizer to tune all learnable parameters in the network, including α and β, aiming to enhance the fidelity of reconstructed images and the overall robustness of the model.

The performance of SBCS-Net will be compared with several current leading compressed sensing reconstruction methods, including Csformer [45], ISTA-Net+, CsNet, AMP-Net, and TransCS, most of which fuse traditional iterative algorithms with deep learning techniques. Performance evaluation is primarily based on objective perceptual metrics of image quality: Peak Signal-to-Noise Ratio (PSNR) and Structural Similarity Index (SSIM). PSNR measures pixel-level reconstruction error. For a ground-truth image X and a reconstructed image $\hat{X}$ of size H×W, it is defined as:

(24) $$\mathrm{PSNR}=10\cdot \log_{10}\left(\frac{MAX^{2}}{\frac{1}{HW}\sum_{i=1}^{H}\sum_{j=1}^{W}{\left(X_{i,j}-{\hat{X}}_{i,j}\right)}^{2}}\right)$$

where MAX is the maximum possible pixel value of the image (1 for our normalized images). SSIM aims to more closely align with human visual perception of image quality by assessing similarities in luminance (*l*), contrast (*c*), and structure (*s*). For two image patches x and y, it is defined as:

(25) $$\mathrm{SSIM}\left(x,y\right)=\frac{\left(2\mu_{x}\mu_{y}+c_{1}\right)\left(2\sigma_{xy}+c_{2}\right)}{\left(\mu_{x}^{2}+\mu_{y}^{2}+c_{1}\right)\left(\sigma_{x}^{2}+\sigma_{y}^{2}+c_{2}\right)}$$

where μ and σ represent the mean and variance, and the final score is the average over all image patches. Consequently, SSIM is particularly important for evaluating the structural integrity and visual fidelity of reconstructed images; higher values for both metrics generally indicate better reconstruction quality. All comparative models are obtained from their publicly available source code or projects and tested using their default recommended configurations. To ensure fairness in comparison, all training images used by the competing methods (if training is required) are consistent with SBCS-Net, originating from the BSD500 dataset. All experiments are conducted on a server equipped with an Intel Xeon 8336 CPU (Santa Clara, CA, USA) and an NVIDIA GeForce RTX 4090 GPU (Santa Clara, CA, USA), using the PyTorch 1.9.0 deep learning framework.

### 4.2. Comparisons

#### 4.2.1. Comparisons with State-of-the-Art Methods

**Figure 3:**
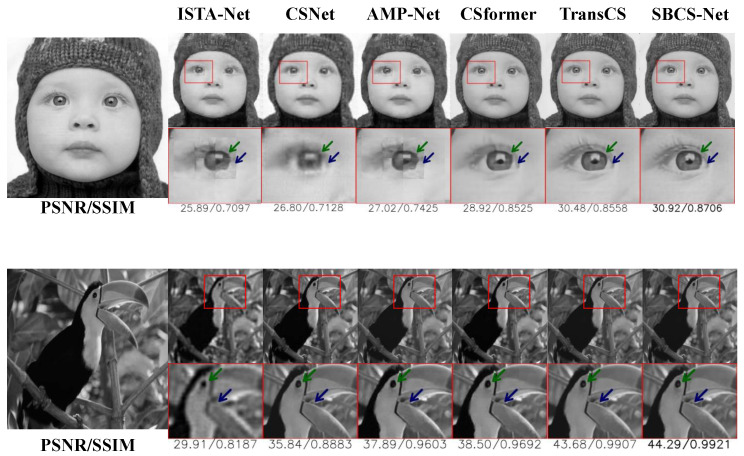
Reconstruction results of the baby and bird images of our SBCS-Net and various competing methods. The sampling rates τ of the first row and second row are 0.04 and 0.25, respectively. Green and blue arrows are used to contrast the details of the two reconstructed images at their respective marked positions. Please zoom in for better comparisons.

In this section, we comprehensively evaluate the proposed SBCS-Net by comparing its performance with state-of-the-art methods, including ISTA-Net, CSNet, CSformer, AMP-Net, and TransCS, on the benchmark datasets Urban100, BSD100, Set5, UCMerced, and BrainImages. We conduct both quantitative and qualitative analyses to demonstrate the superiority of SBCS-Net, using Table 1 for numerical comparisons and Figure 3 for visual assessment.

Table 1 presents a comparison of PSNR and SSIM performance for various SOTA methods—including ISTA-Net, CSNet, CSformer, AMP-Net, TransCS, and our proposed SBCS-Net—across five benchmark datasets (Set5, Urban100, BSD100, UCMerced, and BrainImages) at multiple sampling rates (τ∈{0.04, 0.1, 0.25, 0.3, 0.4, 0.5}). In the results, the best performance at the corresponding sampling rate is marked in red, while the second-best is marked in blue. The experimental results clearly demonstrate the superior performance of SBCS-Net, particularly in terms of reconstruction quality at low sampling rates. Specifically, on the Set5 dataset, SBCS-Net achieves the optimal PSNR and SSIM metrics (marked in red) across all sampling rates. Notably, at challenging low sampling rates (τ=0.04 and τ=0.1), SBCS-Net shows a significant advantage over other methods; at higher sampling rates (τ=0.25,τ=0.3, and τ=0.5), it maintains a leading edge, although close to the second-best methods (marked in blue). For the Urban100 dataset, which contains more complex texture structures, SBCS-Net also exhibits strong performance. Although slightly inferior to CSformer at τ=0.04 and to TransCS at τ=0.1 (marked in blue), it still significantly outperforms other competing methods. Particularly at sampling rates τ=0.25 and τ=0.5, SBCS-Net achieves optimal performance (marked in red), surpassing TransCS in both PSNR and SSIM metrics. On the BSD100 dataset, SBCS-Net demonstrates the most stable performance advantage, achieving optimal results across all sampling rates (all marked in red), fully validating its significant reconstruction stability and effectiveness in handling diverse image complexities. For the UCMerced dataset, SBCS-Net performs best at most sampling rates (marked in red), achieving, for example, 21.38 dB at τ=0.04 compared to CSformer’s 21.06 dB, and 34.35 dB at τ=0.25, surpassing TransCS’s 33.97 dB; it only falls slightly behind TransCS at τ=0.4 (37.98 dB vs. 38.02 dB) but regains the lead at τ=0.5 with 40.18 dB, showcasing its excellent adaptability to the diverse landscape features in remote sensing images. On the BrainImages dataset, SBCS-Net also delivers robust performance, achieving 26.46 dB at τ=0.04 compared to CSformer’s 25.94 dB, and 36.49 dB at τ=0.25, outperforming TransCS’s 35.62 dB; although it trails TransCS slightly at τ=0.1 and τ=0.4 (e.g., 30.17 dB vs. 30.97 dB), it achieves the best performance at τ=0.5 with 40.17 dB, highlighting its robustness in handling high-contrast structures in medical images.

The superiority of SBCS-Net is evident not only in quantitative metrics but also in the visual quality of reconstructed images. Figure 3 shows the reconstruction results for the “Baby” and “Parrot” images at sampling rates τ=0.04 and τ=0.25, respectively. At a sampling rate of τ=0.04, SBCS-Net excels in reconstructing fine details, such as the eyelashes in the “Baby” image, where other methods fail to capture these intricate features, resulting in blurred or distorted regions. SBCS-Net achieves an SSIM value of 0.8706 in this example, significantly higher than comparative methods, and this quantitative result confirms its advantage in maintaining image structure and edge clarity. Similarly, at τ=0.25, SBCS-Net demonstrates its ability to recover complex textures, particularly in the head textures of the “Parrot” image. Its high SSIM value of 0.9921, compared to other methods, also proves from a data perspective that its reconstruction results are structurally closer to the original image with higher fidelity in texture details. In contrast, alternative methods like CSNet and AMP-Net produce reconstructions that are overly smoothed or prone to artifacts, whereas SBCS-Net delivers clear and visually superior results, highlighting its exceptional capability in modeling both local and global image features.

The key to SBCS-Net’s remarkable performance lies in its hybrid architecture, which combines Bayesian sparse estimation with convolutional and attention mechanisms. This integration enables SBCS-Net to effectively capture local details while simultaneously modeling long-range dependencies, a limitation observed in many baseline methods. Furthermore, SBCS-Net demonstrates exceptional robustness at low sampling rates, where traditional and deep learning-based approaches often struggle to maintain reconstruction quality. The combination of quantitative excellence and superior visual fidelity positions SBCS-Net as a significant advancement in the field of compressed sensing reconstruction, offering a robust and efficient solution for high-quality image recovery.

#### 4.2.2. Statistical Significance Analysis

To further validate the performance advantages of SBCS-Net over other advanced methods from a statistical perspective and to assess the stability and reliability of its results, we conducted additional statistical evaluations. This includes a detailed per-sampling-rate performance comparison with representative SOTA methods on the UCMerced dataset, as well as the introduction of statistical ranking for multi-algorithm comparison.

**Figure 4:**
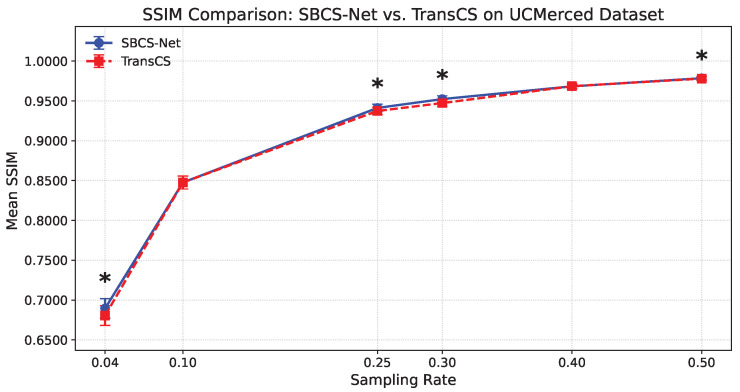
Mean SSIM comparison on UCMerced. * Indicates SBCS-Net significantly outperforms TransCS (Wilcoxon one-sided test, p<0.05).

**Figure 5:**
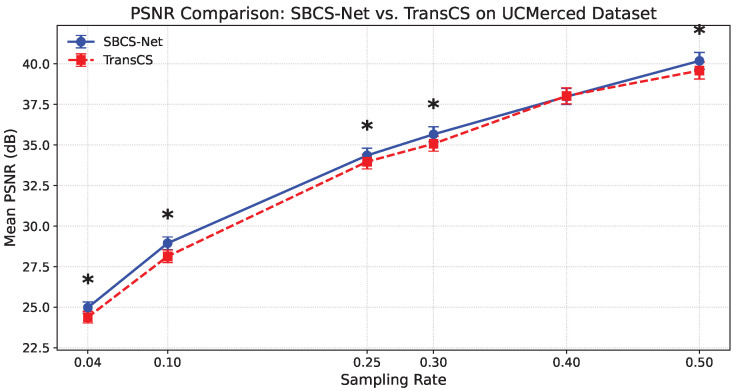
Mean PSNR comparison on UCMerced. * Indicates SBCS-Net significantly outperforms TransCS (Wilcoxon one-sided test, p<0.05).

We first conducted a meticulous comparison of the reconstruction performance of SBCS-Net against the recently high-performing TransCS method at different sampling rates on the UCMerced remote sensing image dataset. Figure 4 and Figure 5, respectively, show the comparison results of the two methods in terms of Mean SSIM and Mean PSNR (dB). As clearly visible in the figures, each data point for SBCS-Net (blue solid line) and TransCS (red dashed line) is accompanied by error bars. These error bars visually represent the distribution of results from multiple independent experiments, reflecting the stability of the reconstruction performance.

In the figures, we marked with an asterisk (*) instances where SBCS-Net’s performance is statistically significantly superior to TransCS. As shown in Figure 4, at sampling rates of 0.04, 0.25, 0.30, and 0.50, the SSIM values of SBCS-Net were demonstrated to be significantly higher than those of TransCS via the Wilcoxon one-sided test (p<0.05). Similarly, it can be observed from Figure 5 that at all tested sampling rates, the PSNR values of SBCS-Net were statistically significantly superior to those of TransCS (Wilcoxon one-sided test, p<0.05). These statistical test results from pairwise comparisons provide strong statistical support for the performance advantage of SBCS-Net over TransCS on the UCMerced dataset.

To evaluate the performance of SBCS-Net among a broader range of SOTA methods and to perform statistical ranking, we employed Critical Difference (CD) Diagrams for analysis. This method, typically based on the Friedman test followed by post hoc multiple comparison tests (such as the Nemenyi test), visually presents the average rankings of multiple algorithms under different conditions and the statistically significant differences between them.

**Figure 6:**
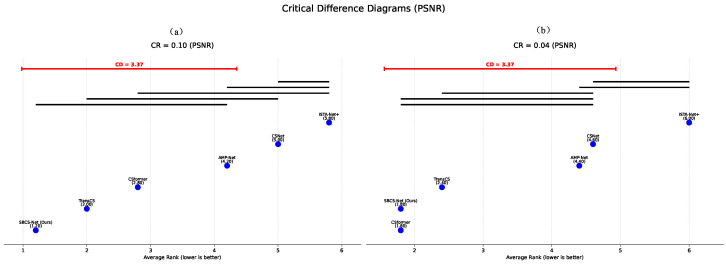
Critical difference diagrams for PSNR performance of SBCS-Net against SOTA methods at different CR. The diagram shows average ranks. Algorithms connected by a thick black line do not have statistically significant performance differences. The CD value is 3.37. (**a**) Results at CR = 0.10. (**b**) Results at CR = 0.04.

Figure 6 shows the comparison of average rankings on the PSNR metric for SBCS-Net against several mainstream compressed sensing reconstruction algorithms, including TransCS, CSformer, AMP-Net, CSNet, and ISTA-Net+, at compression/sampling rates (CRs) of 0.10 and 0.04, respectively. In the diagram, a lower average rank indicates better performance. The Critical Difference value (CD = 3.37) is marked on the diagram. If the difference in average ranks between two algorithms exceeds this CD value, their performance difference is considered statistically significant; conversely, if algorithms are connected by the same thick black line, the performance difference between them is not statistically significant.

As can be seen from Figure 6a (CR = 0.10), SBCS-Net achieved the lowest average rank (1.20) and is not connected to other methods by a thick black line, indicating a significant performance advantage over most comparative methods at this sampling rate. Under the more challenging low sampling rate condition of CR = 0.04 (Figure 6b), SBCS-Net (Ours) and CSformer both have an average rank of 1.80, with no statistically significant difference between them. However, they are both significantly superior to TransCS (2.40) and the lower-ranked AMP-Net, CSNet, and ISTA-Net+.

### 4.3. Noise Robustness Analysis

**Figure 7:**
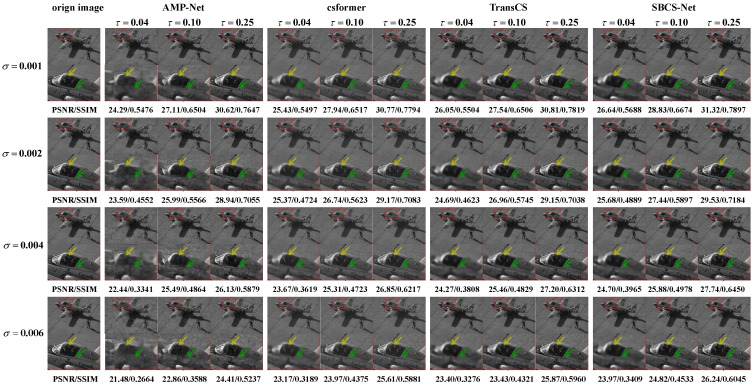
Visual comparisons for various image CS methods on airplane image from dataset BSD100 at different sampling rates τ∈{0.04, 0.10, 0.25}. The first, second, and third rows correspond to the original image with Gaussian noise of multi-level variances (σ∈{0.001, 0.002, 0.004, 0.006}), respectively.

Robustness under various noise levels is a crucial metric for evaluating the reliability of CS reconstruction algorithms. To analyze the noise robustness of SBCS-Net, we conducted experiments under different Gaussian noise levels (σ∈{0.001, 0.002, 0.004, 0.006}) and sampling rates (τ∈{0.04, 0.10, 0.25, 0.30}). The quantitative results are shown in Table 2, and qualitative visual comparisons are presented in Figure 7.

Table 2 indicates that SBCS-Net consistently achieves the highest PSNR and SSIM values across all Gaussian noise levels and sampling rates, highlighting its superior reconstruction capability. At a Gaussian noise level of σ=0.004 and a sampling rate of τ=0.30, SBCS-Net achieves a PSNR of 26.75 dB and an SSIM of 0.7399, outperforming other methods such as AMP-Net (25.68 dB, 0.7138) and Csformer (25.79 dB, 0.7151). As the Gaussian noise level increases, SBCS-Net’s performance gradually degrades but maintains a significant performance advantage over competing methods. Similarly, Table 3 evaluates SBCS-Net’s performance under salt-and-pepper noise, showing consistent superiority across noise levels σ∈{0.02, 0.05, 0.10} and sampling rates τ∈{0.04, 0.10, 0.25, 0.30}. For instance, at a salt-and-pepper noise level of σ=0.05 and τ=0.30, SBCS-Net achieves a PSNR of 19.84 dB and an SSIM of 0.6209, surpassing TransCS (19.54 dB, 0.6159) and Csformer (18.27 dB, 0.6093). Even at a higher noise level of σ=0.10, SBCS-Net maintains its lead, with a PSNR of 17.04 dB and an SSIM of 0.5591 at τ=0.30, compared to TransCS’s 16.99 dB and 0.5523. This robust performance under both Gaussian and salt-and-pepper noise underscores SBCS-Net’s strong adaptability to different noise types, making it highly suitable for practical applications where noise characteristics vary.

The key to SBCS-Net’s effective handling of different noise types lies in the adaptive capability of its Bayesian sparse estimation module and the synergistic processing mechanism of CNN and Transformer. For Gaussian noise, SBCS-Net’s Bayesian sparse estimation module utilizes its inherent Gaussian noise probability model, as elucidated in Equation (1), and achieves precise statistical modeling of Gaussian noise through the end-to-end learned noise precision parameter β, thereby realizing effective probabilistic signal separation. Subsequently, the CNN and Transformer modules collaboratively perform deep optimization on the output of the Bayesian estimation, including smoothing residual noise, sharpening image details, and strengthening the overall structure. For non-Gaussian impulse noise such as salt-and-pepper noise, the processing mechanism has a different emphasis: although the Gaussian noise assumption of the Bayesian module does not perfectly match the characteristics of impulse noise, it can still perform crucial preliminary regularization on the signal through end-to-end learned parameters like noise precision β and sparsity precision α. It adaptively adjusts its reliance on severely corrupted data items and utilizes sparse priors to help suppress large isolated outliers, thus providing a cleaner input basis for subsequent deep learning modules to process impulse noise. On this foundation, the core impulse noise removal is primarily accomplished by the deep learning modules: the CNN module, through data-driven learning, can accurately identify and repair local, high-magnitude salt-and-pepper noise points; meanwhile, the Transformer module, with its global context modeling capability, maintains the overall image structure and long-range dependencies while the CNN performs local restoration, preventing the introduction of secondary distortions. It is this sophisticated division of labor and close collaboration among the modules that endows SBCS-Net with its excellent robustness in complex noise environments.

Figure 7 displays the qualitative reconstruction results of various comparative methods under the influence of different intensities of Gaussian noise, further visually substantiating the robustness of SBCS-Net. Taking a typically adverse condition as an example, i.e., a Gaussian noise level of σ=0.004 and a sampling rate of τ=0.25, SBCS-Net demonstrates significant reconstruction advantages: it not only visually succeeds in reconstructing many intricate details of aircraft structural components, such as wing edges and fuselage textures—markedly superior to the severely degraded or blurred reconstruction effects in other comparative methods—but also, corresponding to this visual improvement, SBCS-Net achieves an SSIM value of 0.6450 under this condition, notably higher than AMP-Net’s 0.5879, Csformer’s 0.6217, and TransCS’s 0.6312. This higher SSIM value quantitatively proves that SBCS-Net’s reconstruction results are structurally more similar to the original image, thereby better preserving image structural integrity and perceptual quality. These excellent visual and quantitative results intuitively reflect SBCS-Net’s capability, as described above, to effectively recover high-quality images from various noisy measurements through the adaptive noise modeling of its Bayesian sparse estimation module and the synergistic action of the CNN and Transformer modules.

The experimental results demonstrate that SBCS-Net’s theoretical foundation—the Bayesian reconstruction algorithm—combined with its hybrid deep architecture, can provide robust reconstruction under different noise conditions. This makes it particularly suitable for practical applications in noisy environments such as medical imaging and remote sensing, where measurement noise is inevitable and reconstruction reliability is paramount. Therefore, SBCS-Net’s ability to maintain excellent reconstruction quality even at higher noise levels fully validates the effectiveness of its core approach of fusing Bayesian theory with a deep learning architecture to enhance the robustness of compressed sensing.

### 4.4. Complexity Analysis

**Figure 8:**
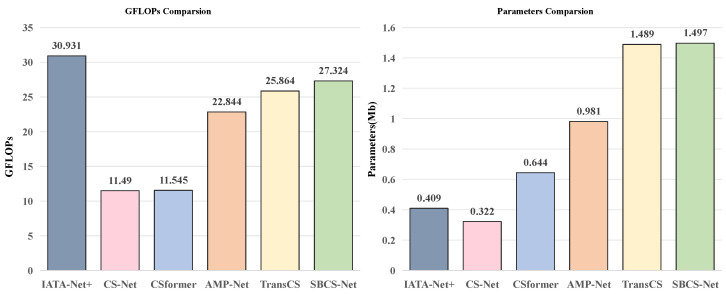
Comparison of the number of GFLOPs and parameters required to run a 256×256 pixel image in the model and the number of model parameters for τ=0.1.

The computational complexity and parameter efficiency of SBCS-Net were evaluated and compared with other SOTA methods, with specific results presented in Figure 8 and Table 4. Figure 8 shows that when processing a 256×256 pixel image, SBCS-Net’s computational cost (27.324 GFLOPs) and parameter count (1.497 MB) are slightly higher than some models such as CSformer (11.545 GFLOPs, 0.644 MB) and TransCS (25.864 GFLOPs, 1.489 MB). Similarly, the GPU time consumption data in Table 4 indicate that SBCS-Net’s average inference time (e.g., approximately 0.0495 s at τ=0.50) is also slightly higher than some comparative methods. However, as revealed by the experimental results, this modest increase in computational resources yields significant improvements in reconstruction quality, robustness, and adaptability to complex signals.

From a theoretical complexity perspective, SBCS-Net effectively controls computational demands through its modular design detailed in Section 3: its core Bayesian sparse estimation module, implemented via neural networks and image block-wise processing, significantly alleviates the large-scale matrix operation bottlenecks common in traditional BCS methods; the CNN modules (such as Pre-block and Post-block) inherently possess efficient local feature processing capabilities; and the computational overhead of the Transformer module is also managed through sequence input based on image patches and a fixed network depth. Therefore, although SBCS-Net has a higher theoretical complexity than simpler models due to the deep integration of multiple complex components, its overall computational load remains within an acceptable range, achieving a balance between performance and efficiency through end-to-end optimization.

This trade-off between performance and computational cost is particularly crucial for large-scale sensor network applications. It is worth noting that SBCS-Net follows a strategy of lightweight front-end sampling and cloud-based execution of complex reconstruction. Its slightly higher computational overhead at the reconstruction end is intended to achieve significant advantages in reconstruction quality and robustness under challenging conditions such as low sampling rates and high noise. This implies that sensor networks can recover high-value information from fewer or lower-quality measurements, which not only promises to enhance the overall operational efficiency of the network but also boosts the system’s reliability and application potential in complex and variable environments, providing a higher-quality data foundation for subsequent precise analysis and intelligent decision-making.

Therefore, the increased complexity of SBCS-Net is not without purpose. By enhancing reconstruction quality and robustness, it allows for more aggressive compression strategies at the resource-constrained sensor end, thereby optimizing the end-to-end efficiency of the entire sensor network. This strategy of shifting computational pressure to the backend and empowering lightweight front ends by improving reconstruction algorithm performance holds significant practical importance for processing large-scale sensor network data. Although there is an increase in computation time, considering its performance advantages under challenging conditions like low sampling rates and high noise, this trade-off is reasonable and beneficial for many sensor network applications that pursue high precision and reliability.

### 4.5. Ablation Studies

**Figure 9:**
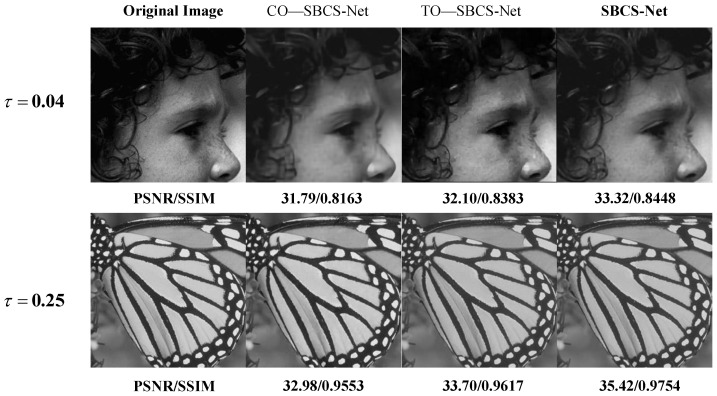
Visual comparison of reconstructed images under different ablation settings (CO–SBCS-Net, TO–SBCS-Net, and SBCS-Net) for τ=0.04 and τ=0.25 on BSD100 dataset.

To evaluate the importance of each component in SBCS-Net, we conducted systematic ablation experiments on the BSD100 dataset at different sampling rates. All experimental variants retained the fundamental Bayesian sparse estimation module. Building upon this, two main ablated variants were constructed and compared with the complete SBCS-Net: the first is TO-SBCS-Net, which retains the Bayesian sparse estimation module and the Transformer-based global feature modeling module but removes the specialized CNN modules, Pre-block and Post-block, to examine performance in the absence of CNN’s fine-grained local feature processing; the second is CO-SBCS-Net, which retains the Bayesian sparse estimation module and the Pre-block and Post-block CNN modules but excludes the core Transformer module (encoder and decoder), aiming to assess reconstruction effectiveness without the global contextual understanding provided by the Transformer. Table 5 and Figure 9 present the quantitative and qualitative results of these experiments, respectively.

As shown in Table 5, we compared CO-SBCS-Net (CNN-only, combined with Bayesian sparse estimation), TO-SBCS-Net (Transformer-only, combined with Bayesian sparse estimation), and the complete SBCS-Net on the BSD100 dataset at sampling rates τ=0.10, 0.25, 0.30 in terms of PSNR and CPU/GPU runtime. The experimental data show that SBCS-Net, integrating CNN and Transformer, fully leverages the local feature extraction capabilities of CNN and the global modeling advantages of Transformer. Its PSNR values (27.76 dB, 31.54 dB, and 32.79 dB, respectively) are significantly superior to those of CO-SBCS-Net (26.42 dB, 30.19 dB, 30.97 dB) and TO-SBCS-Net (26.53 dB, 30.43 dB, 31.18 dB), with improvements of up to 1.82 dB. It is noteworthy that despite the significant performance improvement of SBCS-Net, the increase in computational overhead is relatively limited. SBCS-Net’s GPU runtime (0.0492–0.0495 s) is only 12–19% higher than that of CO-SBCS-Net (0.0413–0.0414 s) and TO-SBCS-Net (0.0439–0.0442 s), while its CPU time (0.221–0.235 s), although increased compared to the latter two (0.118–0.134 s and 0.109–0.119 s, respectively), remains within an acceptable range, indicating that the combined architecture achieves a good balance between performance and efficiency.

Furthermore, Table 6 investigates the impact of different network depths on model performance. On the BSD100 dataset, under conditions of varying layer counts (two, four, and six layers) and sampling rates (τ=0.04, 0.10, 0.25), SBCS-Net consistently outperforms the two ablated variants. Particularly at six layers, SBCS-Net achieves optimal performance (PSNRs of 29.32 dB, 33.17 dB, and 37.61 dB, respectively), markedly superior to the four-layer configuration (28.59 dB, 32.83 dB, and 37.26 dB) and the two-layer configuration (24.38 dB, 30.29 dB, and 31.49 dB). Concurrently, the six-layer SBCS-Net also surpasses CO-SBCS-Net (27.84 dB, 32.08 dB, and 37.13 dB) and TO-SBCS-Net (28.37 dB, 32.67 dB, and 37.22 dB) of equivalent depth, confirming that a deeper architecture more effectively enhances image reconstruction quality.

The qualitative results in Figure 9 further validate the aforementioned quantitative analysis. Moreover, SSIM, as a key visual quality metric for structural similarity, more clearly reveals the contribution of each component to the fidelity of the reconstructed image. Under low sampling rate conditions (e.g., τ=0.04), SBCS-Net exhibits distinct advantages in edge preservation and detail reconstruction compared to the ablated variants. Images generated by TO-SBCS-Net present blurred textures, primarily due to the lack of the CNN module’s capability for fine-grained capture and enhancement of local high-frequency details. Conversely, CO-SBCS-Net shows insufficient global coherence in reconstructed images, directly reflecting the model’s loss of effective modeling capability for long-range dependencies and global context after the removal of the Transformer module, thereby affecting the accurate recovery of the overall structure. When the sampling rate is increased to τ=0.25, SBCS-Net, by effectively combining the local feature extraction of CNN and the global modeling power of Transformer, generates images that are visually much sharper and more structurally faithful than those from the ablated variants. These visual improvements are highly consistent with the higher SSIM values for SBCS-Net (potentially reported in Table 5 or an associated table). These higher SSIM values indicate that SBCS-Net’s reconstructions are closer to the original images in terms of luminance, contrast, and key structural information, resulting in more natural visual effects and fewer artifacts. This again emphasizes the comprehensive advantage of its complete architecture in restoring fine image structures and enhancing overall visual quality.

These comprehensive results highlight the complementary roles of the CNN and Transformer modules in SBCS-Net: the CNN module specializes in capturing local textures and fine details, while the Transformer module enhances global consistency and robustness. The integration of these complementary components enables SBCS-Net to achieve its current state-of-the-art performance level, fully validating the effectiveness of its architectural design and its superiority in the field of compressed sensing image reconstruction, especially in generating reconstructed images with high structural fidelity and visual quality.

### 4.6. Comparison with Traditional Compressed Sensing Algorithms

To further highlight the advantages of the proposed SBCS-Net framework in terms of image reconstruction performance and computational efficiency, this section provides a direct comparison with two representative traditional compressed sensing algorithms: OMP and BCS. OMP, as a classic greedy algorithm, is widely used for its simplicity and speed. BCS, on the other hand, is a probability model-based reconstruction method that offers a more complete theoretical framework for signal recovery, but its traditional implementations typically involve high computational complexity.

Under identical experimental conditions, we evaluated these three methods based on GPU runtime, PSNR, structural SSIM, and MSE. The specific comparison results are shown in Table 7.

In terms of computational efficiency, SBCS-Net demonstrates a significant advantage. Its GPU runtime is 0.86 s, comparable to the fast OMP algorithm (0.9 s), and considerably lower than the 16.06 s required by traditional BCS. This indicates that SBCS-Net successfully controls computational overhead while utilizing neural networks for complex reconstruction, endowing it with practical application potential. This also proves that SBCS-Net addresses the issue of high computational cost in BCS algorithms. Traditional BCS often involves large-scale matrix inversion operations when solving for the posterior distribution (such as the covariance matrix calculation shown in Equation (6), with complexity up to $O(N^{3})$), leading to high computational costs and difficulty in scaling to large signals. SBCS-Net, by implementing the Bayesian sparse estimation module within a neural network framework and typically processing at the image block level, significantly reduces the need for direct large-scale matrix operations. Furthermore, its end-to-end learning approach avoids the time-consuming iterative optimization processes used for hyperparameter estimation in traditional BCS, thereby substantially improving computational efficiency.

SBCS-Net also achieves substantial advantages in various objective evaluation metrics for reconstructed images (PSNR, SSIM, MSE).

## 5. Conclusions and Outlook

This paper successfully proposes and validates SBCS-Net. This network innovatively fuses the core mechanisms of SBL with an iterative deep learning architecture incorporating CNN and Transformer. Through end-to-end learning of key SBL parameters, SBCS-Net not only effectively overcomes the challenges of manual hyperparameter tuning and some computational bottlenecks inherent in traditional Bayesian methods but also fully leverages the powerful local feature extraction capabilities of CNN and the superior global context modeling advantages of Transformer, achieving a deep synergy between model-driven and data-driven approaches.

Extensive benchmark tests and comparative experimental results clearly demonstrate that SBCS-Net exhibits reconstruction accuracy and visual quality superior to existing mainstream compressed sensing methods across multiple public datasets, particularly under demanding conditions including extremely low sampling rates and strong noise. Notably, the model also demonstrates excellent stability and leading reconstruction performance on various out-of-distribution datasets with significant distributional differences, encompassing remote sensing imagery, medical images, and complex urban scenes. This strongly proves its good generalization capability and practical adaptability to different signal types, thereby providing an efficient and robust new paradigm for signal recovery in resource-constrained sensor networks.

Despite the significant progress achieved by SBCS-Net, its deployment scalability for ultra-large-scale networks and its computational efficiency in meeting extreme real-time processing requirements present areas for further improvement. Concurrently, while the model’s effectiveness has been validated on various image-based data, its direct applicability and optimal performance on a broader range of sensor data types beyond images, such as high-dimensional time-series signals or complex video streams, warrant deeper investigation and targeted optimization.

Looking ahead, our research will focus on continuously enhancing the practicality and performance boundaries of SBCS-Net. Key efforts will explore more effective model lightweighting techniques, distributed deployment architectures, and co-optimization with specialized hardware to improve its operational efficacy in large-scale and real-time scenarios. Concurrently, we aim to extend the core framework of SBCS-Net to more diverse sensor data modalities. Furthermore, incorporating insights from and fusing with other advanced learning paradigms—including online learning, continual learning, and adaptive transfer learning—will be an important direction to enhance the model’s adaptability to dynamically changing environments and its long-term application stability, ultimately aiming to promote greater value from SBCS-Net in complex and variable real-world sensor network applications.

## Figures and Tables

**Table 1:** PSNR (dB) and SSIM comparisons of different methods on Urban100, BSD100, Set5, UCMerced, and BrainImages datasets at multiple sampling rates τ∈{0.01, 0.04, 0.1, 0.25, 0.3, 0.4, 0.5}. Red color in the table footer indicates the optimal values, and blue color indicates the sub-optimal values.

Datasets	Methods	0.01		0.04		0.10		0.25		0.30		0.40		0.50	
		PSNR	SSIM	PSNR	SSIM	PSNR	SSIM	PSNR	SSIM	PSNR	SSIM	PSNR	SSIM	PSNR	SSIM
Set5	ISTA-Net+ (CVPR2018)	20.25	0.5608	23.42	0.6287	28.47	0.8309	34.02	0.9188	35.38	0.9397	37.44	0.9573	39.25	0.9689
	CSNet (TIP2019)	20.15	0.5447	27.12	0.7988	31.07	0.8925	35.89	0.9473	37.25	0.9473	38.91	0.9611	40.74	0.9691
	CSformer (TIP2023)	21.84	0.5892	29.27	0.8239	33.04	0.9243	37.04	0.9583	38.44	0.9614	40.62	0.9723	42.37	0.9793
	AMP-Net (TIP2021)	20.45	0.5563	27.25	0.8065	31.43	0.8977	36.25	0.9514	37.82	0.9583	39.55	0.9694	41.48	0.9756
	TransCS (TIP2022)	22.98	0.6287	29.02	0.8215	32.74	0.9197	37.26	0.9652	38.53	0.9693	41.40	0.9773	42.42	0.9852
	SBCS-Net (Ours)	**23.50**	**0.6305**	**29.32**	**0.8252**	**33.17**	**0.9252**	**37.61**	**0.9658**	**38.74**	**0.9712**	**41.81**	**0.9796**	**42.72**	**0.9858**
UCMerced	ISTA-Net+ (CVPR2018)	17.52	0.4294	22.23	0.6138	25.65	0.7751	26.44	0.8859	31.43	0.9173	33.69	0.9383	36.71	0.9568
	CSNet (TIP2019)	19.47	0.4473	24.16	0.6485	26.92	0.8059	28.93	0.9174	33.25	0.9258	34.79	0.9578	38.97	0.9695
	CSformer (TIP2023)	21.06	0.4793	24.52	0.6821	27.89	0.8396	33.72	0.9329	34.21	0.9418	37.19	0.9605	39.33	0.9712
	AMP-Net (TIP2021)	19.23	0.4416	23.87	0.6543	27.64	0.8147	29.92	0.9238	34.17	0.9349	35.68	0.9691	39.46	0.9783
	TransCS (TIP2022)	20.82	0.4769	24.39	0.6807	28.14	0.8409	33.97	0.9374	35.07	0.9473	**38.02**	**0.9685**	39.58	0.9780
	SBCS-Net (Ours)	**21.38**	**0.4830**	**24.97**	**0.6895**	**28.94**	**0.8476**	**34.35**	**0.9412**	**35.66**	**0.9523**	37.98	0.9683	**40.18**	**0.9785**
BrainImages	ISTA-Net+ (CVPR2018)	22.59	0.6591	26.95	0.7852	29.24	0.8471	32.09	0.8893	33.29	0.9134	34.92	0.9287	36.63	0.9517
	CSNet (TIP2019)	24.23	0.6962	29.73	0.8237	31.53	0.8842	34.49	0.9257	36.71	0.9403	37.08	0.9411	38.37	0.9512
	CSformer (TIP2023)	25.94	0.7046	30.49	0.8421	32.87	0.8997	35.43	0.9368	36.88	0.9523	37.59	0.9576	39.42	0.9711
	AMP-Net (TIP2021)	24.35	0.6982	28.47	0.8296	31.94	0.8863	34.72	0.9293	36.84	0.9437	37.62	0.9423	38.89	0.9568
	TransCS (TIP2022)	25.83	0.7032	**30.97**	**0.8546**	33.11	**0.9082**	35.62	0.9426	**38.05**	0.9572	**38.89**	0.9672	40.09	0.9715
	SBCS-Net (Ours)	**26.46**	**0.7165**	30.17	0.8306	**33.17**	0.9025	**36.49**	**0.9483**	37.31	**0.9653**	38.70	**0.9676**	**40.17**	**0.9730**
Urban100	ISTA-Net+ (CVPR2018)	15.23	0.4127	19.65	0.5351	23.44	0.7165	28.78	0.8825	30.04	0.9061	32.32	0.9373	34.36	0.9571
	CSNet (TIP2019)	15.89	0.4438	19.69	0.5973	23.17	0.7789	28.76	0.9033	29.52	0.9278	32.83	0.9462	33.47	0.9627
	CSformer (TIP2023)	18.92	0.4893	**22.57**	**0.6781**	25.27	0.8213	30.57	0.9287	31.07	0.9313	33.21	0.9575	34.23	0.9721
	AMP-Net (TIP2021)	17.84	0.4562	20.74	0.6013	23.90	0.7859	29.55	0.9174	30.44	0.9317	33.26	0.9521	34.65	0.9702
	TransCS (TIP2022)	**19.53**	**0.5104**	22.30	0.6756	**25.87**	**0.8347**	30.46	0.9234	**31.47**	**0.9446**	**33.49**	**0.9621**	34.58	0.9735
	SBCS-Net (Ours)	19.43	0.4925	21.79	0.6671	25.43	0.8254	**30.78**	**0.9297**	31.21	0.9372	33.34	0.9583	**34.93**	**0.9763**
BSD100	ISTA-Net+ (CVPR2018)	17.45	0.4234	22.21	0.5397	24.89	0.6837	28.83	0.8379	29.92	0.8673	31.77	0.9063	33.52	0.9357
	CSNet (TIP2019)	18.23	0.4576	23.77	0.6497	26.31	0.7714	30.04	0.8997	30.69	0.9135	32.94	0.9299	34.96	0.9478
	CSformer (TIP2023)	22.42	0.4892	24.96	0.6709	26.54	0.7749	30.75	0.9022	31.54	0.9189	34.21	0.9443	35.91	0.9589
	AMP-Net (TIP2021)	18.67	0.4623	24.04	0.6537	26.16	0.7688	30.13	0.9002	30.88	0.9142	33.24	0.9379	35.43	0.9517
	TransCS (TIP2022)	22.26	0.4848	24.68	0.6637	27.60	0.7953	31.45	0.9047	32.27	0.9215	34.52	0.9499	36.52	0.9671
	SBCS-Net (Ours)	**22.53**	**0.5062**	**25.12**	**0.6716**	**27.76**	**0.7982**	**31.54**	**0.9124**	**32.79**	**0.9237**	**34.96**	**0.9522**	**36.89**	**0.9688**

**Table 2:** PSNR (dB) and SSIM on BSD100 with Gaussian noise at various levels σ and sampling rates τ. Red color in the table footer indicates the optimal values, and blue color indicates the sub-optimal values.

σ	τ	AMP-Net (TIP2021)		Csformer (TIP2023)		TransCS (TIP2022)		SBCS-Net	
		PSNR	SSIM	PSNR	SSIM	PSNR	SSIM	PSNR	SSIM
0.001	0.04	23.28	0.5475	24.17	0.5683	23.76	0.5578	24.48	0.5824
	0.10	25.32	0.6558	26.29	0.6872	25.53	0.6814	26.72	0.6933
	0.25	27.73	0.7930	28.24	0.8013	28.68	0.8087	29.24	0.8267
	0.30	28.37	0.8007	28.97	0.8279	29.23	0.8316	29.62	0.8422
0.002	0.04	22.59	0.4879	23.21	0.4957	23.46	0.5083	23.67	0.5279
	0.10	24.17	0.6052	25.31	0.6278	24.81	0.6169	25.50	0.6311
	0.25	27.23	0.7583	27.53	0.7597	27.59	0.7618	27.85	0.7746
	0.30	27.64	0.7625	28.05	0.7678	28.17	0.7724	28.31	0.7912
0.004	0.04	20.56	0.4317	21.39	0.4423	21.97	0.4526	22.38	0.4672
	0.10	22.53	0.5289	23.48	0.5413	23.16	0.5326	24.23	0.5583
	0.25	25.41	0.6825	25.54	0.6849	25.97	0.6854	26.21	0.7085
	0.30	25.68	0.7138	25.79	0.7151	26.19	0.7164	26.75	0.7399
0.006	0.04	20.16	0.3859	20.77	0.3912	21.44	0.4079	21.83	0.4179
	0.10	22.76	0.4827	22.77	0.4993	22.79	0.5064	23.41	0.5138
	0.25	24.35	0.6572	24.69	0.6597	25.04	0.6645	25.17	0.6728
	0.30	24.69	0.6917	24.89	0.6983	25.43	0.7034	25.93	0.7174

**Table 3:** PSNR (dB) and SSIM on BSD100 with salt-and-pepper noise at various levels σ and sampling rates τ.Red color in the table footer indicates the optimal values, and blue color indicates the sub - optimal values.

σ	τ	AMP-Net (TIP2021)		Csformer (TIP2023)		TransCS (TIP2022)		SBCS-Net	
		PSNR	SSIM	PSNR	SSIM	PSNR	SSIM	PSNR	SSIM
0.02	0.04	19.05	0.3729	19.93	0.3829	19.87	0.3814	20.01	0.3863
	0.10	19.54	0.4977	20.72	0.5044	20.39	0.5032	20.92	0.5099
	0.25	21.71	0.6783	21.99	0.6837	22.07	0.6845	22.70	0.6864
	0.30	22.14	0.7175	22.10	0.7195	23.09	0.7214	23.29	0.7250
0.05	0.04	15.08	0.2219	16.93	0.2331	17.18	0.2385	17.35	0.2405
	0.10	16.59	0.3422	17.51	0.3591	17.46	0.3579	17.95	0.3609
	0.25	18.13	0.5490	18.52	0.5604	19.04	0.5601	19.34	0.5690
	0.30	18.27	0.6018	18.27	0.6093	19.54	0.6159	19.84	0.6209
0.10	0.04	13.27	0.1430	14.73	0.1544	14.72	0.1520	15.00	0.1577
	0.10	14.06	0.2669	15.21	0.2713	15.44	0.2769	15.45	0.2747
	0.25	15.58	0.4776	16.07	0.4959	16.57	0.4943	16.61	0.4996
	0.30	15.93	0.5423	16.54	0.5517	16.99	0.5523	17.04	0.5591

**Table 4:** Time consumption (in seconds) of different methods under various compression rates τ on GPU: RTX 4090.

Methods	GPU Time Consumption (s)					Platform
	τ=0.10	τ=0.25	τ=0.30	τ=0.40	τ=0.50	
ISTA-Net+	0.0227	0.0232	0.0238	0.0241	0.0247	RTX 4090
CSNet	0.0078	0.0084	0.0089	0.0095	0.0099	
CSformer	0.0469	0.0471	0.0476	0.0480	0.0486	
AMP-Net	0.0165	0.0177	0.0181	0.0189	0.0194	
TransCS	0.0241	0.0245	0.0247	0.0251	0.0257	
SBCS-Net	0.0492	0.0494	0.0495	0.0496	0.0499	

**Table 5:** Performance comparison of different methods with various sampling rates.

Methods	τ=0.10			τ=0.25			τ=0.30		
	PSNR	Run Time		PSNR	Run Time		PSNR	Run Time	
		CPU	GPU		CPU	GPU		CPU	GPU
CO-SBCS-Net	26.42	0.118	0.0413	30.19	0.121	0.0413	30.97	0.134	0.0414
TO-SBCS-Net	26.53	0.109	0.0439	30.43	0.115	0.0441	31.18	0.119	0.0442
SBCS-Net	27.76	0.221	0.0492	31.54	0.224	0.0494	32.79	0.235	0.0495

**Table 6:** PSNR comparison with different numbers of layers and sampling rates.

Layer	τ	CO-SBCS-Net	TO-SBCS-Net	SBCS-Net
		PSNR	PSNR	PSNR
2	0.04	22.53	21.07	24.38
	0.10	27.52	26.74	30.29
	0.25	28.17	31.02	31.49
4	0.04	26.46	27.33	28.59
	0.10	30.04	31.59	32.83
	0.25	34.63	35.87	37.26
6	0.04	27.84	28.37	29.32
	0.10	32.08	32.67	33.17
	0.25	37.13	37.22	37.61

**Table 7:** Performance comparison with traditional compressed sensing algorithms.

Method	GPU (s)	PSNR (dB)	SSIM	MES
OMP	0.9	17.21	0.4011	0.0274
BCS	16.06	8.99	0.2666	0.1399
SBCS-Net	0.86	32.73	0.9200	0.0006

## Data Availability

The data presented in this study are available on request from the corresponding author.
